# Does Serial Femtosecond Crystallography Depict State-Specific Catalytic Intermediates of the Oxygen-Evolving Complex?

**DOI:** 10.1021/jacs.3c00489

**Published:** 2023-05-03

**Authors:** Maria Drosou, Gerard Comas-Vilà, Frank Neese, Pedro Salvador, Dimitrios A. Pantazis

**Affiliations:** † Max-Planck-Institut für Kohlenforschung Kaiser-Wilhelm-Platz 1, 45470, Mülheim an der Ruhr, Germany; ‡ Institute of Computational Chemistry and Catalysis, Chemistry Department, University of Girona, Montilivi Campus, Girona, Catalonia 17003, Spain

## Abstract

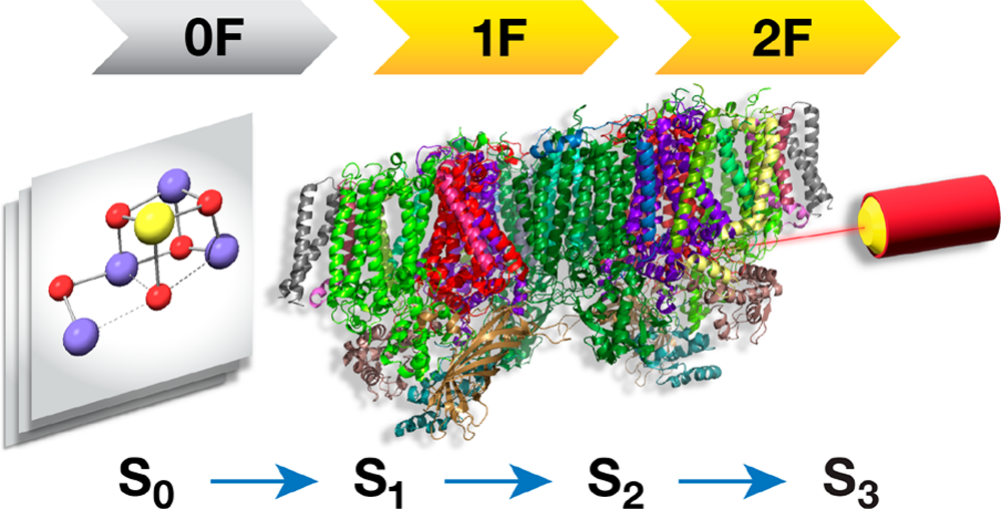

Recent advances in serial femtosecond crystallography (SFX) of photosystem II (PSII), enabled by X-ray free electron lasers (XFEL), provided the first geometric models of distinct intermediates in the catalytic S-state cycle of the oxygen-evolving complex (OEC). These models are obtained by flash-advancing the OEC from the dark-stable state (S_1_) to more oxidized intermediates (S_2_ and S_3_), eventually cycling back to the most reduced S_0_. However, the interpretation of these models is controversial because geometric parameters within the Mn_4_CaO_5_ cluster of the OEC do not exactly match those expected from coordination chemistry for the spectroscopically verified manganese oxidation states of the distinct S-state intermediates. Here we focus on the first catalytic transition, S_1_ → S_2_, which represents a one-electron oxidation of the OEC. Combining geometric and electronic structure criteria, including a novel effective oxidation state approach, we analyze existing 1-flash (1F) SFX-XFEL crystallographic models that should depict the S_2_ state of the OEC. We show that the 1F/S_2_ equivalence is not obvious, because the Mn oxidation states and total unpaired electron counts encoded in these models are not fully consistent with those of a pure S_2_ state and with the nature of the S_1_ → S_2_ transition. Furthermore, the oxidation state definition in two-flashed (2F) structural models is practically impossible to elucidate. Our results advise caution in the extraction of electronic structure information solely from the literal interpretation of crystallographic models and call for re-evaluation of structural and mechanistic interpretations that presume exact correspondence of such models to specific catalytic intermediates of the OEC.

## 1 Introduction

The intimate coupling between electronic and geometric structure is a cornerstone of coordination chemistry. It also underpins all structure–property correlations in transition metal chemistry, because the local electronic structure of a transition metal ion, often distilled in the d-electron configuration and the formal or physical oxidation states,^1−3^ (OSs) cannot be disentangled solely from its coordination geometry. This connection, expressed most succinctly in the parameters of ligand field theory, is more vividly expressed in some transition metal ions than others. Textbook examples of one-to-one correspondence between geometry and electronic structure include distinct isomeric forms for low- versus high-spin electronic configurations, such as the tetrahedral versus square planar conformation of d^8^ complexes. A singularly important case of pronounced stereoelectronic coupling is the Jahn–Teller (JT) effect. In the whole of transition metal chemistry, this is most extreme in the cases of Cu(II) (d^9^) and of high-spin Mn(III) (d^4^). Occupation of the strongly σ-antibonding (d_*z*^2^_) orbital in the latter leads most often to a strong axial elongation of the coordination sphere, clearly identifiable even in heteroleptic complexes (pseudo-JT effect). This is why Mn(III) can typically be unambiguously identified from inspection of geometric parameters alone. At the same time, Mn(III) is clearly distinguishable from the symmetric high-spin Mn(II) (d^5^) and the Mn(IV) (d^3^) ions, the latter having the shortest metal–ligand bonds due to the formal absence of occupied σ-antibonding orbitals.

Manganese ions in the OSs + III and + IV are constituents of one of the most important enzymes of Earth’s biosphere, the photosystem II (PSII) that catalyzes the light-driven oxidation of water into dioxygen.^4−10^ The Mn_4_CaO_5_ cluster at the oxygen-evolving complex (OEC) of PSII cycles through five intermediate states S_*i*_ (*i* = 0–4),^11,12^ storing the oxidizing equivalents required for water oxidation in a progression of alternating electron and proton removals.^13−17^ In the most reduced state (S_0_) the Mn OSs are III–IV–III–III,^18,19^ while in the dark-stable S_1_ state the Mn OSs are III–IV–IV–III. Oxidation of S_1_ leads to the III–IV–IV–IV S_2_ state and further oxidation to S_3_ yields an all-Mn(IV) cluster,^20^ at least as a major population.^21−26^ A final oxidation drives the system through the unobserved—and possibly unobservable—S_4_ state to form and release O_2_, resetting to S_0_. Despite ongoing debates regarding the late steps of the catalytic cycle past the S_2_ state,^9,21^ it is universally accepted that the S_1_ → S_2_ transition involves only a one-electron Mn-centered oxidation. Strong evidence in support of this notion stems from the analysis of X-ray absorption and emission spectroscopies,^6,27−34^ as well as by electron paramagnetic resonance spectroscopy (EPR)^35^ and other magnetic resonance techniques.^18,19,36−51^ The experimental knowledge of the total spin of the OEC in the S_0_ (half-integer ground spin state of *S* = 1/2), S_1_ (integer spin states of *S* ≥ 0), S_2_ (half-integer spin states of *S* ≥ 1/2), and S_3_ (integer spin states of *S* ≥ 3) intermediates, in combination with the known Mn OSs,^16,52^ provides precise values for the total number of unpaired electrons in the OEC, while additional information from hyperfine spectroscopies^18−20,41,48,53−55^ combined with quantum chemical calculations^16,20,45,46,49,56−64^ often uniquely identifies the positions of individual Mn ions within the cluster.

The local electronic structures of Mn(III) and Mn(IV) ions are reflected not only in their spectroscopic properties but also in their local coordination spheres, as established from rich and century-spanning coordination chemistry and crystallography. Therefore, it could be expected that crystallographic characterization of the OEC poised in distinct S-states of the catalytic cycle would also reveal the Mn valence distribution within the OEC and its state-to-state changes. In reality, protein crystallography of PSII only recently has made significant progress in this direction. Starting from the first low-resolution crystallographic models of PSII in 2001,^65^ obtained with conventional synchrotron radiation sources, the field has moved through gradual improvements^66−70^ to the current era of X-ray free-electron laser (XFEL) sources, targeting the nominal S_1_ state.^71,72^ The advent of serial femtosecond crystallography (SFX) has more recently enabled attempts at characterizing models for the S_2_ (1-flash, 1F), S_3_ (2F), and S_0_ (3F) intermediates.^73−84^ Discrepancies among SFX-XFEL studies^77−79,81^ and an apparent lack of consistency with spectroscopic OSs,^21^ has fueled ongoing debates about the nature of the highly contested S_3_ state and about the mechanism of O–O bond formation itself.^22,23,81,85−91^ For the most part, such debates arise when a new crystallographic model is taken at face value, making the explicit assumption that it is a precise depiction of a given catalytic intermediate, often disregarding standard stereoelectronic correlations of Mn(III/IV) chemistry and state-specific information derived from spectroscopy. Recent studies, however, call into question the reliability and information content of such crystallographic models,^92−94^ suggesting that the above assumption is unjustified.

In the present work we focus first on the S_1_ → S_2_ transition, which represents an incontrovertible one-electron oxidation of a Mn ion. This step represents the maximal possible conversion among the transitions in the OEC cycle and is unique in that it does not involve proton transfer or water insertion that would cause significant structural alterations. Significantly, despite the heterogeneity demonstrated by EPR studies in both the S_1_ and S_2_ states, all suggested models possess distinct metal oxidation states—specifically, two Mn(III) and two Mn(IV) ions in the S_1_ state and one Mn(III) and three Mn(IV) ions in the S_2_ state. We address the question whether SFX-XFEL models depict the correct Mn OSs and adequately capture structural changes at the OEC in this catalytic step, or in other words whether the existing SFX-XFEL 1-flash models actually depict the S_2_ state of the OEC or not. We use a comprehensive array of methods, ranging from structure-based analysis and Mn(III) distortion metrics to evaluation of electronic structure and magnetic/spectroscopic properties. Among others we employ an effective oxidation state analysis applied for the first time to the OEC. The computed OSs for the XFEL models are compared to those of computational models of the OEC, which are used solely as representative examples of structures that align with spectroscopic parameters. Our results highlight severe limitations and occasional complete breakdowns in the connection between geometry and electronic structure for the available crystallographic models of the 1F (“S_2_”), but also of the 0F (“S_1_”) states. The available models appear to be over-reduced; they do not clearly reflect one-electron oxidation in the S_1_ → S_2_ transition, and they do not feature well-defined Mn OSs or electron counts. These fundamental problems and the discrepancies observed between PSII monomers suggest that the existing SFX-XFEL models should not be used as a basis to extract conclusion concerning electronic structure features. Given that this is the simplest possible catalytic step, the ability of current SFX-XFEL models to describe more complicated states or transitions is expected to be more limited, as analyzed here for the case of the two-flash (2F) structures that would nominally describe the S_3_ state of the OEC. Therefore, at their present stage of development such models should be used with great care for addressing questions of state-specific structural changes, structural heterogeneity, or mechanism, for a system as complex as the OEC.

## 2 Methodology

**Figure 1:**
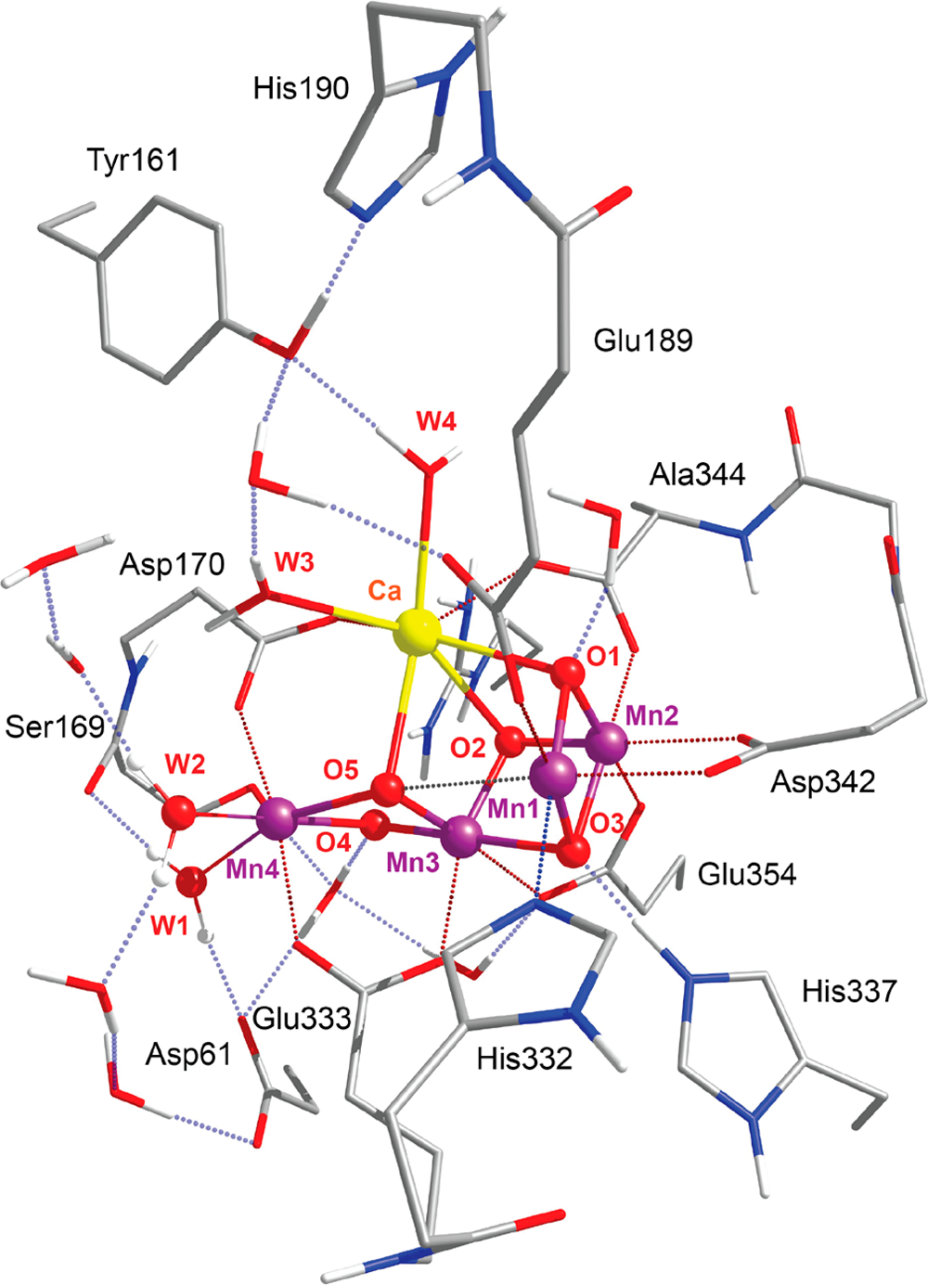
Inorganic core of the OEC from the **6JLJ**(81) XFEL model, monomer **A**, with the amino acid residues that are included in the models used in this study. Hydrogen atoms attached to carbon atoms are omitted for clarity.

Models of the OEC were constructed from the following XFEL structures: **6JLJ** (0F), **6JLK** (1F), and **6JLL** (2F);^81^**7COU** (0F) and **7CJJ** (1F);^84^**6W1O** (0F), **6W1P** (1F), and **6W1V** (2F);^79^**6DHE** (0F), **6DHF** (1F), and **6DHO** (2F).^82^ The first two sets will be occasionally referred to in this work as “Okayama” models, and the last two sets, as “Berkeley” models. Each model includes the Mn_4_CaO_5_ cluster, the amino acid residues Asp61, Ser169, Asp170, His332, Glu333, His337, Asp342, Glu189, His190, Ala344, and Tyr161 from the D1 protein subunit and Glu354 and Arg357 from the CP43 protein subunit, the terminal water-derived ligands W1–W4, and eight crystallographic water molecules (Figure 1).

All quantum chemical calculations were carried out with Orca 5.^95^ Geometry optimization was performed only for the hydrogen atoms using the BP86 functional^96,97^ along with the zeroth-order regular approximation to the exact relativistic Hamiltonian (ZORA)^98−100^ and the ZORA-recontracted^101^ def2-TZVP(-f) basis sets.^102^ For the calculation of the pairwise Mn–Mn exchange coupling constants and the ^55^Mn and ^14^N hyperfine coupling tensors, the TPSSh^103^ functional was employed. The broken-symmetry methodology for the calculation of exchange coupling constants, spin states, and projected hyperfine coupling constants has been described in previous papers.^45−47,49,57,58,104^ The ZORA-def2-SVP basis sets were used for H and C, and ZORA-def2-TZVP(-f), for N, O, Ca, and Mn.^102^ For the calculation of the ^55^Mn and ^14^N hyperfine coupling tensors, the ZORA-def2-TZVP(-f) basis set was used with decontracted s-functions and three additional tight s-functions obtained by scaling the innermost exponent of the original basis by 2.5, 6.25, and 15.625.^105^ The chain-of-spheres (RIJCOSX) approximation^106^ to exact exchange was employed, to reduce computational time. The SARC/J auxiliary basis sets^107^ were used for the Coulomb fitting. “Picture-change” effects due to the use of the scalar relativistic Hamiltonian were also taken into account. Dense integration grids (DefGrid3 in Orca convention) were used throughout. The mean-field (SOMF) approximation to the Breit–Pauli operator (SOCType 3) was used for the treatment of spin–orbit coupling. The potential was constructed to include one-electron terms, to compute the Coulomb terms using the RI approximation, to incorporate exchange via one-center exact integrals including the spin-other orbit interaction, and to not include local DFT correlation (SOCFlags 1,3,3,0 in ORCA).

The bond valence sum (BVS) of each Mn ion was calculated using the equation:
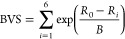
1where *R*_*i*_ is the metal–ligand bond length for ligand *i* and parameters *R*_0_ and *B* were derived from a set of well-characterized inorganic crystals by a generalized reduced gradient method.^108^ The parameter values for Mn(III) ions are *R*_0_ = 1.823 Å and *B* = 0.247 Å, and those for Mn(IV) ions are *R*_0_ = 1.750 Å and *B* = 0.374 Å.

Spin-resolved effective fragment orbitals and subsequent effective oxidation states (EOS) analyses were performed with the APOST-3d program^109^ in the framework of the Quantum Theory of Atoms in Molecules (QTAIM) atomic definition based on the lowest energy broken-symmetry TPSSh solutions.^110^ TPSSh has been extensively benchmarked and known to provide the most accurate results for magnetic and spectroscopic properties of Mn complexes.^58,111,112^ We note that the effect of the functional on computed spin densities for the present case is negligible, as for manganese–oxo systems in general. Atomic/fragment overlap matrices in the molecular orbital basis were obtained with Aimall.^113^

## 3 Results and Discussion

### 3.1 Overview of the 0F and 1F SFX-XFEL Models

**Figure 2:**
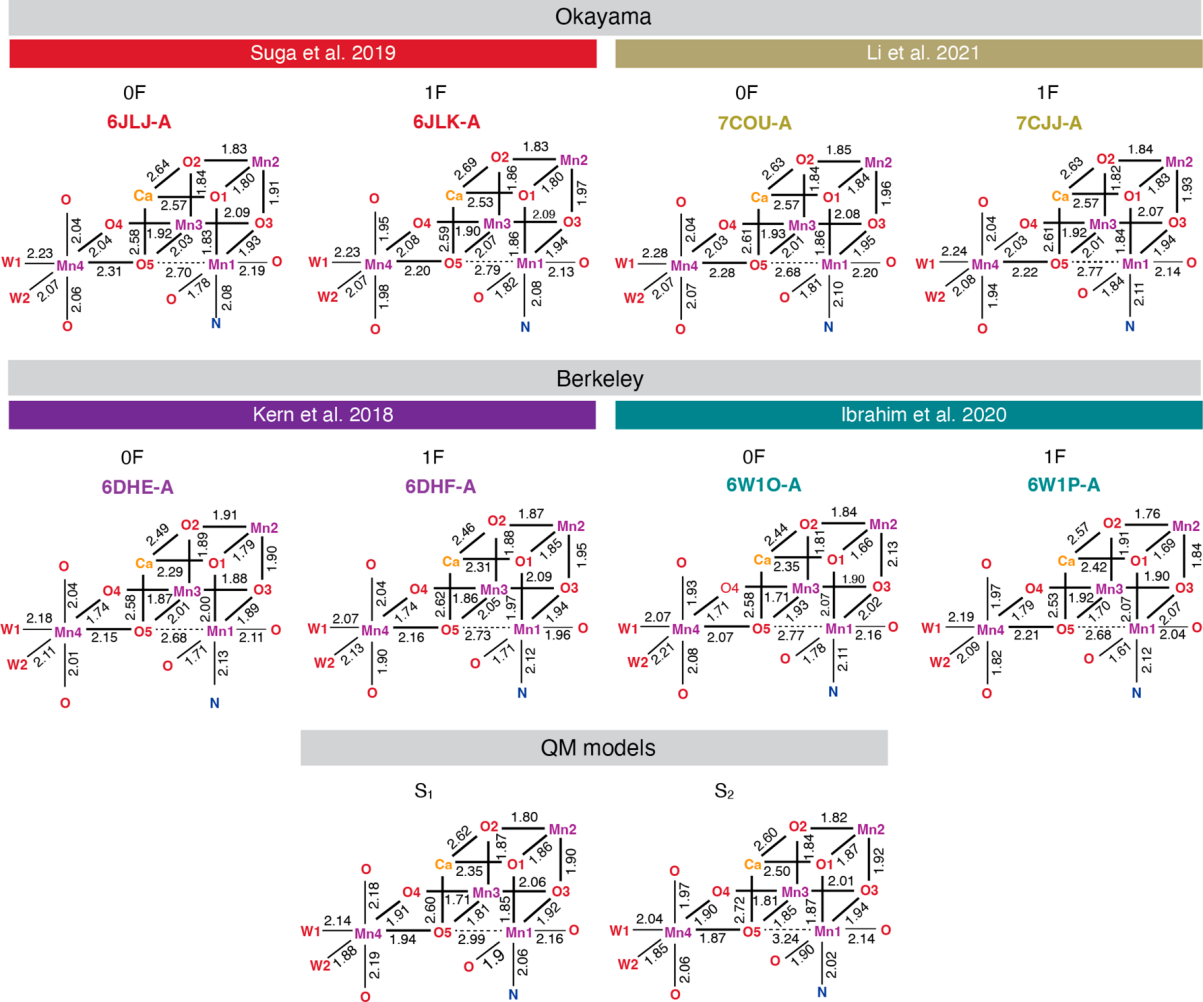
Key bond lengths of XFEL models (monomer A only) and of QM models of the S_1_ and S_2_ states; Okayama models include the **6JLJ** (0F) and **6JLK** (1F) reported by Suga et al.^81^ in 2019, and the **7COU** (0F) and **7CJJ** (1F) reported by Li et al.^84^ in 2021. Berkeley models include the **6DHE** (0F) and **6DHF** (1F) reported by Kern et al. in 2018,^79^ and **6W1O** (0F) and **6W1P** (1F) reported by Ibrahim et al.^82^ in 2020.

We examined the four latest reported SFX-XFEL structures that nominally correspond to the S_1_ (0F) and S_2_ (1F) states of the OEC. The structures **6JLJ** (0F) and **6JLK** (1F) reported by Suga et al.^81^ in 2019, and **7COU** (0F) and **7CJJ** (1F) reported by Li et al.^84^ in 2021 are derived from the PSII of the thermophilic cyanobacterium *Thermosynechococcus vulcanus*. The structures **6DHE** (0F) and **6DHF** (1F) reported by Kern et al. in 2018,^79^ and **6W1O** (0F) and **6W1P** (1F) reported by Ibrahim et al.^82^ in 2020 are derived from the PSII of *Thermosynechococcus elongatus*. In all cases, the crystals were reported as preflashed, followed by dark-adaptation to synchronize all samples. The S_2_ state was populated by illumination of the dark-adapted PSII crystals (0F) with one flash (1F). We examined both monomers (designated A and B) for all crystal structures, since the degree of similarity between them reveals whether the two monomers are synchronized in the same S-state. Key bond lengths of the 0F and 1F XFEL models are shown in Figure 2 for the A monomers and in Figure S1 for the B monomers.

In all XFEL models, Mn1 is axially elongated along the O5–Mn1–Asp342 vector. This distortion is characteristically pronounced with Mn1–O5 distances ranging from 2.68 to 2.79 Å and is observed in all “open-cubane” OEC models of the S_0_–S_2_ states. Mn2 and Mn3 ions do not exhibit pronounced axial elongation, and their coordination sphere comes closer to symmetric octahedral. In all 0F XFEL models, Mn4 is axially elongated along the W1–Mn4–O5 vector. In the 1F structures, Mn4 is axially elongated as well, which creates ambiguity regarding the oxidation of Mn4(III) to Mn4(IV) in the 1F samples.

The same structural parameters of models of the S_1_^61^ and S_2_^16^ states derived from geometry optimization using quantum mechanics (QM) electronic structure methods are also shown at the bottom of Figure 2, for comparison. Notably, all XFEL and QM models examined here can be considered consistent with the distribution of Mn–Mn and Mn–Ca distances as determined by EXAFS spectroscopy.^6,114,115^ The QM models of the S_1_^61^ and S_2_^16^ states are also consistent with EPR spectroscopy. We stress that the QM models are used only as examples of structures consistent with spectroscopic observations and with inherent cleanly defined Mn OSs; structural differences between the XFEL and QM models are not suggested to be an argument for or against the reliability of either. In the QM models, Mn1–O5 distances are larger than in XFEL, while Mn4–O5 distances are smaller. In the QM S_1_ state model, this is because the axial elongation of Mn4 is along the Asp270–Mn4–Glu33 vector; therefore, the Mn4–O5 bond is in an equatorial position. Moreover, contrary to the 1F models, in the QM S_2_ state model the Mn4 ion coordination geometry is very close to symmetric octahedral, consistent with the expected Mn4(IV) OS.

Therefore, a simple visual examination of the 1F XFEL models shows that the Mn4 coordination geometry is more consistent with Mn(III) than with Mn(IV) and suggests that the models may correspond to a lower S-state or a mixture of states. Interestingly, this pattern is visible in all 1F models, along very specific axial directions, and specifically on Mn4, which is Mn(III) in lower S-states. Thus, it cannot be related exclusively to effects arising from resolution limitations or temperature considerations. Ibrahim et al.^82^ estimated standard deviations for the Mn–O bond lengths between 0.09 and 0.25 Å and for the Mn–Mn and Mn–Ca distances between 0.07 and 0.27 Å, and Li et al.^84^ reported that differences in the Mn–Mn and Mn–Ca distances between the 0F and 1F structures were smaller than the experimental resolution. Nevertheless, the Mn–Mn and Mn–Ca distances are consistent with those predicted by EXAFS in both S_1_ and S_2_ states. Hence, even though our analysis cannot explicitly account for the standard deviations in the Mn–ligand distances stemming from limited crystallographic resolution, the axial distortions of the Mn ions in the final XFEL models are a meaningful feature.

The discussion of problematic geometric aspects in crystallographic models of the OEC has a long history. Following the publication of the atomic resolution OEC structure by Umena et al. in 2011,^71^ several studies showed that the proposed model (formally expected to depict the dark-stable S_1_ state) does not correspond to the true S_1_ state of the catalyst, but it rather reflects a mixture of lower OSs.^116,117^ Galstyan et al.^117^ examined what is the precise nature of the photoreduced complex in terms of charge and protonation state, by comparing geometry optimized structures with different charge and protonation states to the X-ray model structure, on the basis of the root-mean-square deviation (RMSD) of the position of the Mn_4_CaO_5_ cluster atoms. Their analysis showed that the experimental structure corresponds to a mixture of highly reduced and catalytically irrelevant states.^117^ Following a similar approach, in Figure S2 we plot the root-mean-square deviations (RMSD) of the Mn_4_CaO_5_ core of the 1F XFEL structures from the QM-optimized S_1_ and S_2_ state models. The plot shows that all Okayama 1F structures (**6JLK** and **7CJJ**), except from **6JLK-B**, are closer to the S_1_ state QM-optimized model than to the S_2_ state QM model, whereas Berkeley structures (**6DHF** and **6W1P**) are more consistent with the QM S_2_ state geometry than the S_1_ state, except from **6W1P-A**. Thus, the structural parameters of most 1F Okayama models and **6W1P-A** imply incomplete S_1_ → S_2_ transition after one-flash illumination.

### 3.2 Mn4 Coordination Sphere in 1F Models

A simple overview of the Mn–O bond lengths in the XFEL models outlined above, suggested that the structural differences between the 0F and the 1F models do not properly reflect one-electron oxidation during the S_1_ → S_2_ transition. Now we focus more closely on the Mn4 ion, because this is identified as the most problematic. As a high-spin d^4^ ion, under octahedral (*O*_*h*_) symmetry Mn(III) has a degenerate ^5^*E*_*g*_ ground state. The Jahn–Teller theorem states that spontaneous geometric distortion of the complex into lower symmetry will create a nondegenerate orbital configuration, typically by axial elongation or compression.^118^ Axial elongation is the typical situation for Mn(III) complexes. The Mn d_*z*^2^_ orbital, which is along the elongated axis of the complex, is stabilized, while the Mn d_*x*^2^–*y*^2^_) orbital is destabilized relative to the symmetric structure. Even in cases of lower symmetry with nondegenerate electronic states, the effect of sufficiently low-lying excited states leads to similar distortions (pseudo-JT effect).

**Figure 3:**
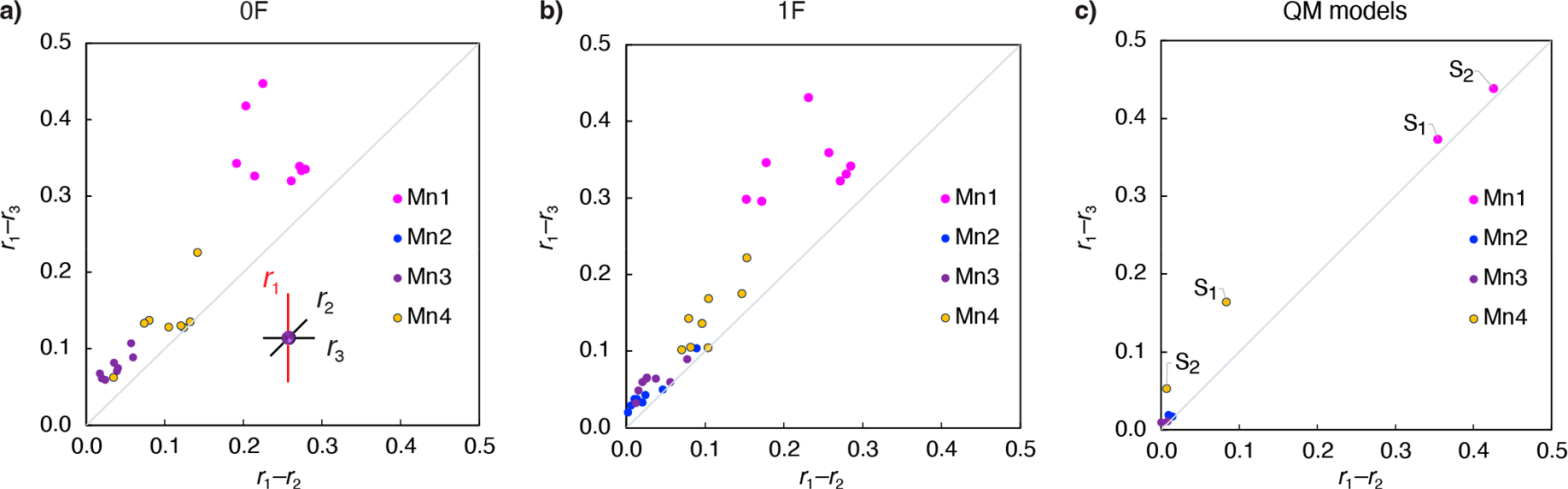
Plots of the axial elongation of Mn atoms in (a) the 0F XFEL models, (b) the 1F XFEL models, and (c) the QM models of the S_1_ and S_2_ states.

Although it can be challenging to define a clear-cut metric for pseudo-JT distortion in complexes with heterogeneous ligand sets because other types of distortion from the octahedral geometry originating in ligand geometric constrains are also present, the differences between the axial and equatorial bond lengths for Mn ions of the XFEL 0F and 1F models, plotted in Figure 3, can be used as descriptors of the axial elongation distortion. The average bond length between Mn and the axial ligands, divided by the value of the parameter *R*_0_ = 1.750 Å from the BVS eq 1, is denoted as *r*_1_, and the average equatorial bond lengths divided by *R*_0_ are denoted as *r*_2_ and *r*_3_. The *axial* ligands are the ligands with the largest average Mn–L bond lengths, i.e. *r*_1_ ≥ *r*_2_ ≥ *r*_3_. The differences between axial and equatorial bond lengths, *r*_1_–*r*_3_ and *r*_1_–*r*_2_, for all Mn atoms in the XFEL structures are given in Table S1. The stronger the axial distortion, the further the ion is found from the (0,0) origin. In the ideal case of axial elongation where the complex symmetry approaches *D*_4*h*_, the equatorial distances *r*_2_ and *r*_3_ are similar; therefore, the point is found close to the diagonal. If a Mn ion had an axially compressed coordination geometry, it would be found near one of the axes.

As shown in Figure 3, Mn1 ions (magenta dots) have a very strong axial distortion due to the large Mn1–O5 distance in the open cubane conformation (Figure 2). Mn2(IV) and Mn3(IV) ions approach the perfect octahedral geometry, i.e. point (0,0), because they have a d^3^ configuration, which is not subject to JT distortion in octahedral complexes. The degree of axial distortion of Mn4 lies between the Mn1 ion and Mn2 and Mn3 ions in both 0F (Figure 3a) and 1F (Figure 3b) states. The similarity of the degree of Mn4 axial elongation between the 0F and 1F models suggests that in the 1F state Mn4(III) is *not* (fully) oxidized to Mn4(IV). The three lowest yellow dots in Figure 3b correspond to **6JKL-B**, **7CJJ-B**, and **6DHF-A** structures. The corresponding diagram for the QM models of the S_1_ and S_2_ states, shown in Figure 3c, can be used for comparison. Unlike in the case of the XFEL models, all Mn2 and Mn3 centers are symmetric, and in the S_2_ state Mn4 is minimally distorted from octahedral symmetry. Therefore, the geometry of the Mn4 coordination sphere in all 1F XFEL models suggests an axially elongated Mn(III) ion rather than a Mn(IV) ion with d^3^ configuration, which implies that Mn4 is not oxidized to Mn(IV) after 1F illumination of the 0F state.

### 3.3 Geometry-Based Oxidation State Analysis

**Figure 4:**
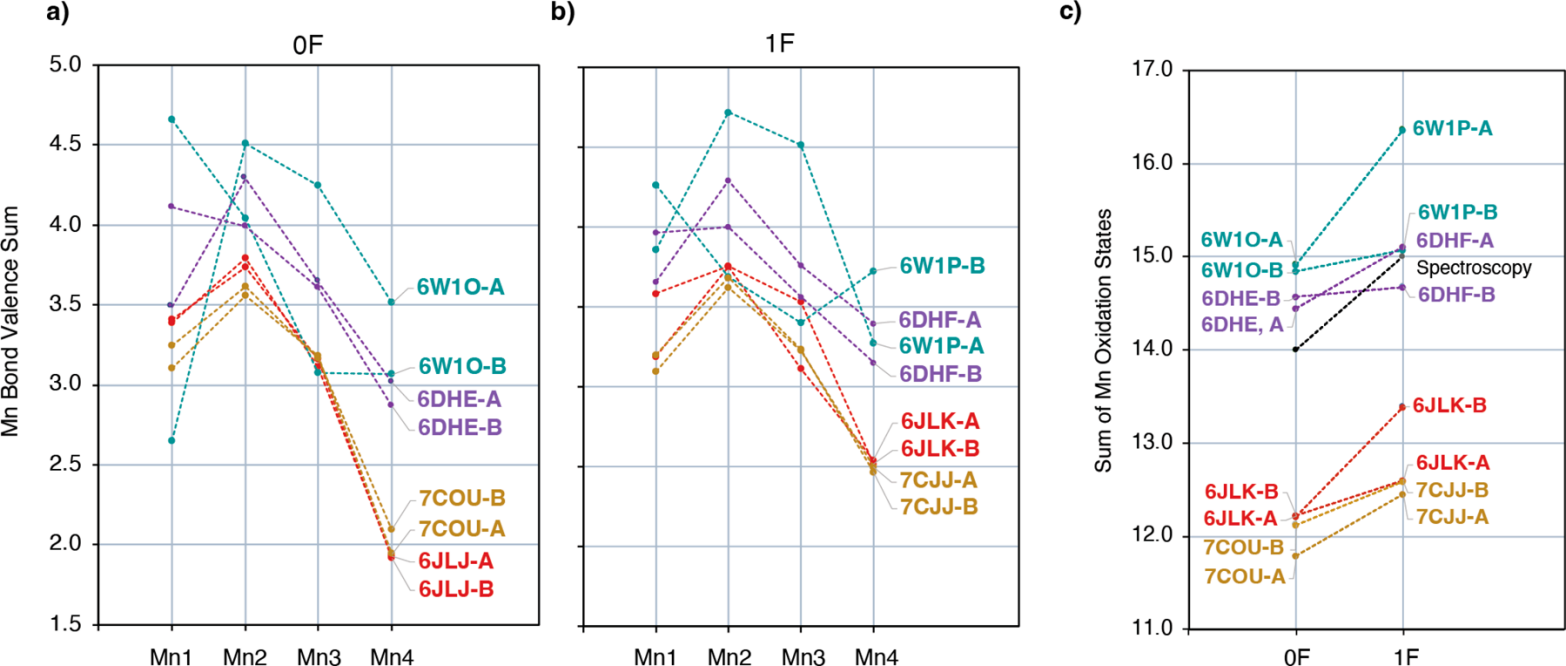
Bond valence sum derived oxidation states for Mn ions of (a) the 0F XFEL structures, (b) the 1F XFEL structures, and (c) sum of Mn oxidation states for the corresponding 0F and 1F structures.

We now approach the problem of determining the Mn OSs of the reported structures using first the bond valence sum (BVS) analysis, a coarse-grained approach that is based on empirical parametrization to correlate the observed metal–ligand bond lengths with the OS of the metal. The BVS analysis has been previously employed to compare the electronic structure of the OEC to synthetic mimics based on Mn OSs.^119,120^ The resulting OSs are shown in Figure 4a for 0F and 4b for 1F models. Detailed values are listed in Table S2. As a confirmation of the consistency of this analysis for the specific system, this approach reproduces the expected Mn OSs for DFT optimized OEC models, with BVS values 2.89, 3.84, 3.78, 2.94 (i.e., close to the formal III–IV–IV–III) for the S_1_ state and 2.93, 3.82, 3.88, 3.74 (III–IV–IV–IV) for the S_2_ state. Very similar results are obtained from other computational models previously reported by different groups (Table S3), confirming that the results do not depend on the choice of the QM method.

The BVS analysis suggests that the Mn OSs of the 0F Okayama models (**6JLJ** and **7COU**) are best described as III–IV–III–II and those of the 1F Okayama models (**6JLK** and **7CJJ**) as III–IV–III–III. In addition, this analysis shows very small differences between the A and B monomers for the Okayama models, which implies that the two monomers are synchronized in the same S-state. The sum of Mn OSs for each structure in the S_1_ and S_2_ states are shown in Figure 4c. Apparently, 1F illumination induces one-electron oxidation of the cluster, but all Okayama structures are two-electron reduced with respect to what is expected based on spectroscopy in the respective state.

The BVS results for the Berkeley models show that Mn coordination geometries are closer to those expected for the respective states compared to the Okayama models, but A and B monomers are not synchronized. This is most prominent in the case of the latest **6W1O** and **6W1P** structures, where the Mn OSs are III–IV–IV–IV and V–IV–III–III, respectively, for the A and B monomers of **6W1O** (0F) and IV–V–IV–III and IV–IV–III–IV, respectively, for the A and B monomers of **6W1P** (1F). In the **6DHE** (0F) and **6DHF** (1F) models, the differences between the two monomers are less pronounced. The Mn OSs are IV–IV–IV–III for both monomers of **6DHE** (0F) and IV–V–IV–III and IV–IV–IV–III for both monomers of **6DHF** (1F). These observations agree with the conclusions of a recent study by Wang et al.,^93^ where analysis of the omit electron density peaks of the Mn ions in the **6JLJ**, **6DHE**, and **6W1O** XFEL demonstrated that the two monomers, A and B, of each structure have different electron density distributions for the Mn ions relative to one another, with the largest deviations observed for the **6W1O** structure. The sum of Mn OSs of the Berkeley models is closer to experiment, as shown in Figure 4c. Notably, in the Berkeley samples, EPR and X-ray emission spectroscopy (XES) measurements were performed in order to detect Mn(II) content at the same time as the diffraction studies, and it was found to be 2%. After one flash only the A monomer seems to be one-electron oxidized among the **6W1P** models as well as among the **6DHF** models.

**Figure 5:**
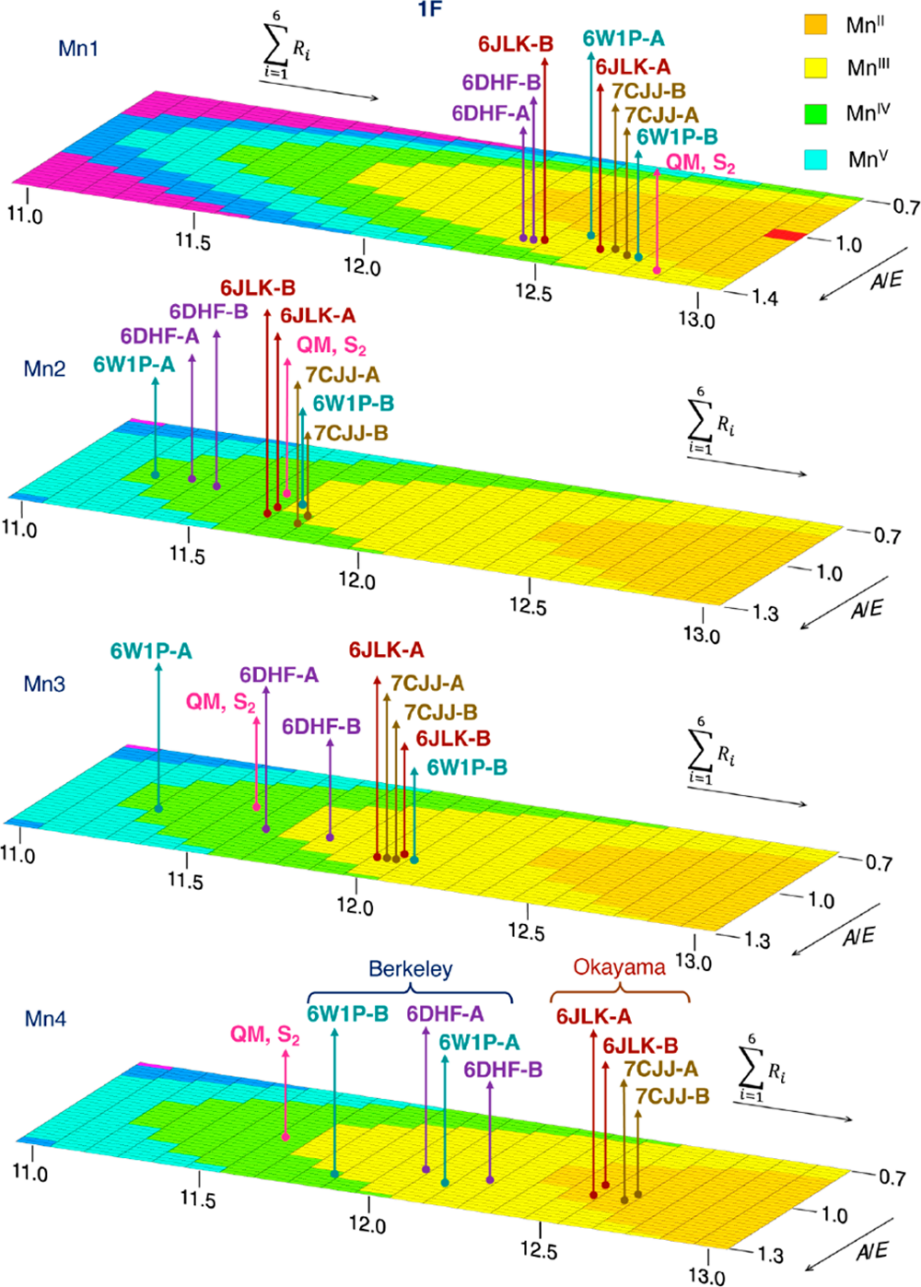
Mn OSs for the 1F XFEL structures derived from bond valence sum analysis using the parameters optimized for Mn(III) on Mn1 and the parameters optimized for Mn(IV) for Mn2, Mn3, and Mn4 ions. Detailed values are given in Table S4. The BVS Mn OSs show that Okayama 1F models are reduced compared to the expected S_2_ state Mn OSs, with Mn3 being Mn(III) and Mn4 being Mn(II). Although Berkeley structures more correctly depict the expected S_2_ state OS distribution, they have a significantly wider spread and monomer-to-monomer heterogeneity. Practically all 1F structures, regardless of their origin (with the marginal exception of the **6W1P-B** monomer) contain a Mn4(III) ion, which in combination with the clear existence of the Mn1(III) ion renders the models inconsistent with the S_2_ state of the OEC.

Two structural parameters can be directly correlated with the Mn OS: (i) the sum of the Mn–ligand bond distances, ∑_*i* = 1_^6^*R*_*i*_, which is discussed above, and (ii) the degree of axial distortion, expressed as the ratio of the averaged axial and equatorial Mn–ligand bond lengths, *A*/*E*. To explicitly address the second point, in Figure 5 we present a refined version of the BVS analysis for the 1F XFEL models. Mn OSs calculated using the BVS method for a range of Mn–O bond lengths are shown. Each row (*A*/*E*) contains the BVS Mn OSs for an octahedral Mn complex with a specific *ratio* of the averaged axial Mn–O bonds to the averaged equatorial Mn–O bonds. Each column corresponds to a specific value of the *sum* of the six Mn–O bonds. The Mn1 OSs calculated using the *R*_0_ and *B* parameters for Mn(III) ions and the Mn2, Mn3, Mn4 OSs calculated using the parameters for the Mn(IV) ions for the 1F XFEL models are shown in different colors, i.e. green for Mn(IV), yellow for Mn(III), and orange for Mn(II). Detailed values are given in Table S4. The Mn OSs of each 1F XFEL structure computed using the BVS method are indicated for each Mn ion of the OEC. For these computations, the four equatorial and two axial Mn–O bond lengths were averaged (Table S5).

Interestingly, the BVS OSs of the Mn ions using average bond lengths fall within a smaller range (Table S6). In all XFEL models, Mn1 and Mn2 have the expected OSs III and IV, respectively. However, Mn3 ions are best described as Mn(III) in all models, except from **6W1P-A** and **6DHF-A**. As already pointed out in Figure 4b, in all structures the Mn4–O bond lengths correspond to either Mn(III) or Mn(II) rather than Mn(IV). Only in **6W1P-B** the Mn4 is Mn(IV), but this is observed only when the Mn(III) parameters are used. Very similar conclusions were reached in a recent study by Amin,^94^ where a definitive correlation between Mn–ligand bond distances and Mn OSs was obtained over a large database of Mn complexes using machine learning based prediction models.

Figure 5 makes evident two distinct structural characteristics of the Okayama versus the Berkeley structures. First, in the Okayama structures the Mn4 ions have larger bond distances on average (right side of the graphs), and second, in the Berkeley structures the axial elongation of the Mn4 ion is more pronounced compared to the Okayama structures (larger *A*/*E* ratio, front side of the graphs). The OSs for the 0F structures using the same method give III–IV–III–II OSs for all Okayama models and III–IV–III–III for all Berkeley models, except **6W1O-A** and **6DHE-A** which are III–IV–IV–III (Figure S3).

From this geometric analysis we conclude that Mn4 is reduced in all examined 1F XFEL models, except **6W1P-B**. Moreover, Mn3 is reduced in all examined 1F XFEL models, except **6W1P-A** and **6DHF-A**. Therefore, none among the 1F structures is consistent with the III–IV–IV–IV Mn OSs distribution expected for the S_2_ state. Among the 0F models, Mn3 is reduced in all Okayama models and in the **6W1O-B** model. Overall, Okayama models are suggested to be reduced to a larger extent and they are shown to not be representative of either the S_1_ or the S_2_ states of the OEC. Among the Berkeley structures, the **6DHE** and **6DHF** models have better synchronized monomers A and B and overall deviate the least from the expected S_1_ and S_2_ states OSs distribution.

### 3.4 Electronic Structure and Spin Densities

**Figure 6:**
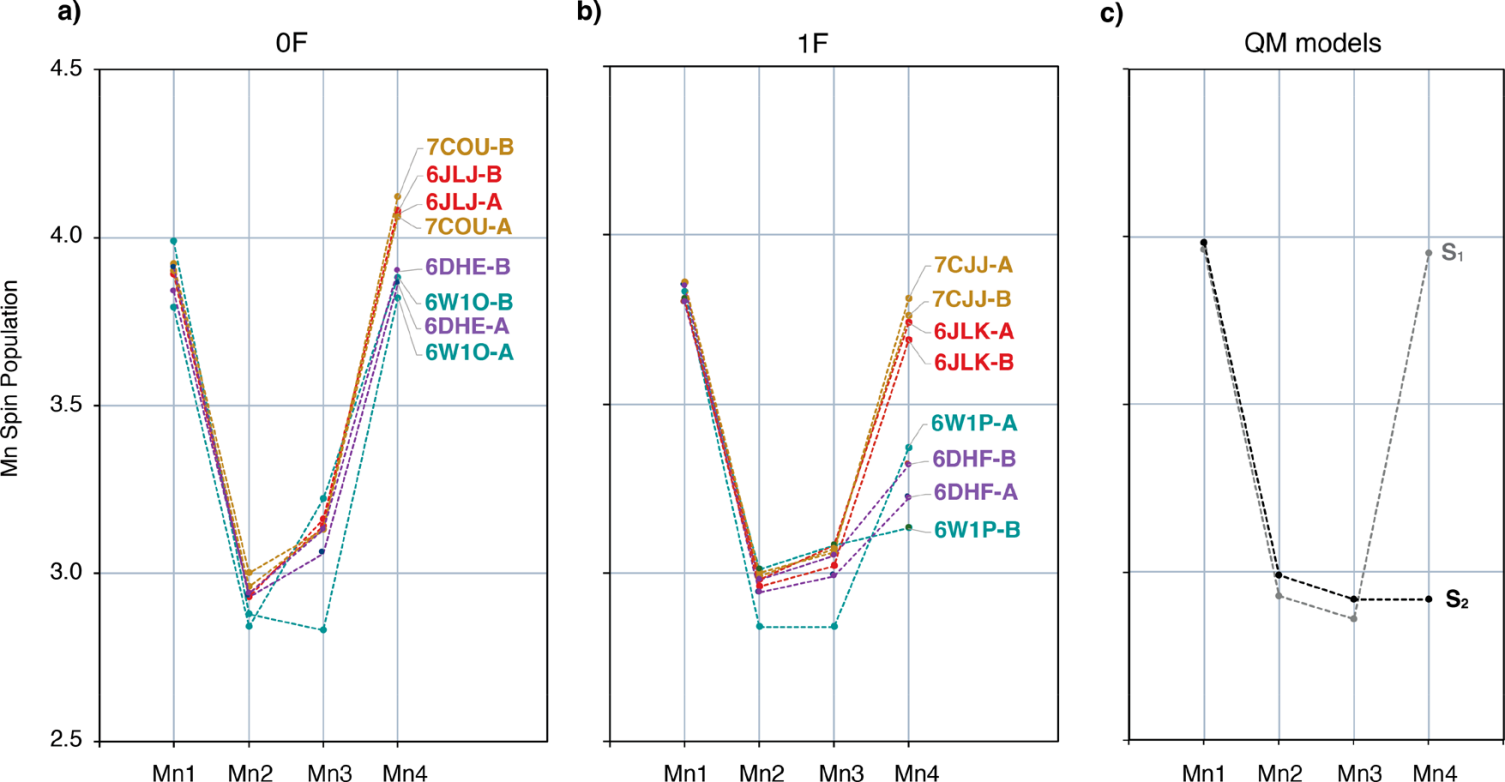
Calculated Mn Mulliken spin populations of (a) 0F XFEL models in the S_1_ state, (b) the 1F XFEL models in the S_2_ state, and (c) the QM models in the S_1_ and S_2_ states.

Following the analysis based exclusively on structural criteria, we now investigate the XFEL models with respect to their electronic structure calculated using density functional theory (DFT). The electron density of each 0F structure was calculated using the charge and spin multiplicity that correspond to the S_1_ state in its lowest energy broken-symmetry state (α–β–α–β for Mn1–Mn2–Mn3–Mn4 and multiplicity 1) and that of each 1F structure was calculated using the charge and spin multiplicity that correspond to the S_2_ state (α–β–β–α for Mn1–Mn2–Mn3–Mn4 and multiplicity 2), in order to study the spin density distribution among the atoms of the Mn_4_CaO_5_ cluster. The absolute values of the derived Mulliken spin populations are plotted in Figure 6, and detailed values are given in Table S7. The QTAIM Mn spin populations are given in Table S8 and plotted in Figure S4. The calculated Mn spin populations of the models derived from the dark-adapted (0F) crystals are consistent with Mn OSs III–IV–IV–III expected for the S_1_ state in all cases. However, Mn3 spin population is higher than in the QM S_1_ model for all 0F XFEL structures, except **6W1O-A**, indicating that Mn3 is reduced to Mn3(III), in agreement with the BVS analysis (Figure S2).

Among the 1F XFEL models, the spin population of Mn4 for all Okayama models (**6JLK** and **7CJJ**) is close to 3.7, leading to Mn OSs of III–IV–IV–III, similar to those of the S_1_ state, instead of III–IV–IV–IV expected for the S_2_ state. The spin population of Mn4 for all Berkeley models (**6W1P** and **6DHF**) is near 3.3, intermediate between the Okayama XFEL models and the QM S_2_ state model, which indicates that the Mn4 geometry in these structures is not clearly consistent with either Mn4(IV) or Mn4(III). The spin population of Mn3 in the 1F models is higher than in the case of the QM S_2_ state model, as seen also in the 0F models.

The Okayama models **6JLJ** and **7COU** of the 0F samples, as well as the **6JLK** and **7CJJ** of the 1F samples, are very similar. The similarity between the A and B monomers of each structure suggests that the monomers are synchronized in the same S-state. However, the DFT analysis suggests that in the Berkeley models, especially the **6W1O** and **6W1P**, the electronic structure of the Mn ions is different among the A and B monomers.

To summarize, spin population analysis shows that in the 1F structures Mn4 is Mn(III) instead of Mn(IV), and in all the XFEL structures Mn3 ions, except from **6W1O-A** and **6W1P-A**, are possibly reduced to Mn(III). Notably, the computed spin populations of the **6DHE-A** and **6DHF-A** models are closest to those of the S_1_- and S_2_-state QM models, respectively.

### 3.5 Effective Oxidation State Analysis

Spin populations account for the *average* excess of alpha electrons on the atoms, which can be misleading when it comes to OS assignment.^121^ The OS is based on a winner-takes-all principle for the electrons involved in bonds, rather than on average quantities. Hence, specific methods for OS assignment treat electrons individually. We now approach the problem of OSs assignment for the reported XFEL models using—for the first time in the case of the OEC—the effective oxidation state (EOS) analysis.^121^ The EOS is a general approach to extract formal OSs from a given wave function. The molecular system is first divided into fragments, which typically correspond to the metal centers and the ligands. EOS analysis relies on Mayer’s spin-resolved effective fragment orbitals (EFOs), i.e. the natural orbitals of the net fragment’s density, and their occupations. The spin-resolved EFOs are sorted by decreasing occupation number and the electrons (electron pairs in closed-shell descriptions) are assigned to the most occupied EFOs until the total number of electrons is reached, thus obtaining an effective configuration of the atoms/ligands within the molecule. The OS of each fragment considered is then obtained by subtraction from the corresponding nuclear charges. Importantly, the difference in occupation from the last occupied (LO) and first unoccupied (FU) EFOs in the EOS procedure can be used to assess to which extent the electron distribution can be represented by the formal ionic model of the OS. The reliability of the overall assignment can thus be quantified using the index *R*(%) defined as
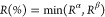
2where *R*^σ^ (%) = 100 min (1, max(0,λ_*LO*_^σ^ – λ_*FU*_^σ^ + 1/2)). *R* can take values from 50%, the worst-case scenario, with frontier EFOs degenerate in occupancy, to 100% when the OS assignment is considered undisputable, i.e. when the difference in occupation of the frontier EFOs exceeds half-electron. Experience indicates that *R*(%) values around 60–70% are typically obtained in complicated bonding situations. Therefore, the value of *R* reflects the uncertainty on the overall OSs assignment and can be used as a criterion to detect problematic structural models of Mn clusters. For example, two crystal structures of the same dinuclear Mn(III)Mn(IV) complex shown in Figure S5, one of which has structurally distinguishable Mn(III) and Mn(IV) ions, while the other does not allow crystallographic distinction between the Mn(III) and Mn(IV) coordination sites due to statistical disorder,^122^ give the anticipated OSs together *R*(%) values of 81.8 and 57.3, respectively.

We applied the EOS analysis to assess the most plausible OS assignment for the Mn ions of the XFEL models. We examined the 0F models with the charge and spin multiplicity that correspond to the S_1_ state as well as to those of the S_0_ state, considering the possibility that the samples can be (at least partially) one-electron reduced compared to the formal dark-stable S_1_ state. We then examined the 1F structures with the charge and spin multiplicity that correspond to the S_2_ state, as well as to the S_1_ and S_0_ states, to examine the possibility that the sample is one- or two-electron reduced. The *R*(%) was used as a criterion for the viability of the formal Mn OSs that correspond to each state. Since the protonation states of the terminal W2 ligand on Mn4 are still under debate,^123^ no assumption was made and different protonation states were considered (Table S9); the protonation states that give the largest (best) *R*(%) values are used for the results reported in Table 1. In all cases we defined up to 15 fragments for EOS analysis, namely the four Mn1–Mn4 centers, five O1–O5 moieties (in some cases protonated), four W1–W4 water ligands (in some cases deprotonated), the Ca nucleus, and the remaining external coordination sphere (amino-acid residues and crystallographic water molecules).

**Table 1:** *R*(%) Values for the EOS Calculated Using Combinations of Charge and Multiplicity That Correspond to Different S-States for the 0F and 1F XFEL Structures^a^

	0F								QM
	**6JLJ-A**	**6JLJ-B**	**7COU-A**	**7COU-B**	**6DHE-A**	**6DHE-B**	**6W1O-A**	**6W1O-B**	S_1_
S_0_ (III, IV, III, III)	**77.6**	**75.4**	**72.2**	**82.7**	**81.0**	**81.8**	76.9	**81.2**	70.4
S_1_ (III, IV, IV, III)	60.8	63.5	63.7	66.4	65.9	59.6	**77.0**	<50^b^	**79.2**

	1F								QM
	**6JLK-A**	**6JLK-B**	**7CJJ-A**	**7CJJ-B**	**6DHF-A**	**6DHF-B**	**6W1P-A**	**6W1P-B**	S_2_
S_0_ (III, IV, III, III)	**81.1**	70.8	**79.9**	**77.7**	**84.5**	**83.5**	72.7	**79.0**	<50^c^
S_1_ (III, IV, IV, III)	61.0	**75.0**	64.7	71.2	69.7	64.4	**80.5**	66.9	50.2
S_2_ (III, IV, IV, IV)	51.7	58.0	56.5	58.4	66.3	64.6	68.4	71.5	**78.4**

a When EOS assignation does not correspond to the nominal values for the Mn center, the *R* value is below 50%.

b EOS assignment leads to Mn3(III) and oxyl O5(−1).

c EOS assignment leads to Mn2(III) and Mn4(IV).

First of all, in almost all cases EOS analysis yields the expected OSs for all fragments, i.e., Ca (+2), neutral water ligands, and O(−2) or OH(−1), depending on the protonation state, as well as the OS of the Mn centers. Hence, in Table 1 and Tables S8 and S9 from the Supporting Information (SI) we report only the OS of the Mn centers and the corresponding *R*(%) index of the assignation. When the assignation is other than the formally expected (e.g., in the case of **6W1O–B** in the S_1_ state) an *R* < 50% is reported.

For all 0F structures, significantly higher *R*(%) values are obtained when the total charge and spin multiplicity of the S_0_ state are used instead of the S_1_ state. Only in the case of **6W1O-A**, the *R*(%) values are almost the same for both states. This suggests that the 0F XFEL structural models are more consistent with an S_0_ state assignment rather than with the dark-stable S_1_ state they are supposed to depict. Notably, the QM model of the S_1_ state is described by a higher *R*(%) value with the total charge and spin multiplicity of the S_1_ state than those of the S_0_ state. The occupation numbers of the frontier EFOs, given in Table S10, provide additional insight into the electronic structure reasons behind this result. The LO EFO corresponds in all cases to a p-type EFO on an oxo moiety, while the FU sits on a Mn center. In the favorable cases, the occupation of the LO on the oxo fragment is around 0.7, while that of the FU on a Mn center is around 0.4. This leads to a pretty clear assignation with *R*(%) values around 70–80%. For the 0F structures with the charge and multiplicity of the S_1_ state, the low *R*(%) values are associated with a “problematic” Mn3–O5 bond, which is far from ideal. As identified also from the BVS analysis (Figure S3) the geometry of Mn3 best corresponds to Mn(III) rather than Mn(IV). The consequence revealed by the EOS analysis is that occupation of the FU EFO on Mn3 increases to almost 0.5, while that of LO on O5 decreases to ca. 0.6. One can thus argue that O5 is partially oxidized (i.e., it has a somewhat higher oxyl character). This result regarding the nature of Mn3 is important because this is precisely the additional center that is present as Mn(III) in the S_0_ state compared to the S_1_. Therefore, the present analysis likely depicts a genuine reversion of the 0F structures to the S_0_ state, i.e. reduction of the sample, or an unexpectedly high initial population of the S_0_ state in the dark-adapted samples to begin with.

Focusing now on the 1F structures, the EOS analysis shows that if the charge and multiplicity of the S_2_ state are assumed, the assignment of Mn OSs that correspond to the S_2_ state (i.e., III–IV–IV–IV) is not achieved for several models (see, e.g., **6JLK** or **7CJJ** in Table S9). The deprotonation of W2 shows some improvement, but still the *R*(%) values of the assignations are rather small, with the largest value computed for the monomer **6W1P-B** (71.5%). The reason is a very high occupation (higher than 0.5 in some cases) of a d-type EFO on the formal Mn4(IV), which is partially (or fully) reduced. Notably, the *R*(%) values for all Berkeley 1F structures range between 64.6 and 71.5, exceeding those of the Okayama structures that range from 51.7 to 58.4. By contrast, the QM model of the S_2_ state is more consistent, giving the largest *R*(%) values when the charge and multiplicity of the S_2_ state is imposed. We should note at this point that other reported^124−126^ QM models of the S_2_ state give similar *R*(%) values (Table S11), which further supports the validity of our approach and demonstrates that QM-optimized models of the OEC provide an inherently clean correspondence between structure and OS regardless of the assumptions made in the construction of a given computational structural model and the method used for optimizing it. Assuming the charge and spin multiplicity of the S_1_ state for the 1F models leads to some improvement in terms of *R*(%) values, albeit not consistently across the board. In this case the models **6JLK-B** and **6WP1-A** provide the highest *R*(%) values for the S_1_ state charge and multiplicity, suggesting that these models are most consistent with the dark-adapted Mn OSs as opposed to their “1F” nature. However, the most remarkable conclusion regarding the 1F XFEL models and the EOS analysis is that the electronic structure of the S_0_ state again provides the best fit for most of them.

The above analysis from an electronic structure perspective leads to the conclusion that the XFEL models depict states of the OEC that are reduced beyond the expected physiological OSs of the Kok cycle, both in the 0F and in the 1F series.

### 3.6 Magnetic and Spectroscopic Properties

The above analysis provided solid results which show that the 1F XFEL models are not in general representative of the S_2_ state of the OEC, a conclusion that holds particularly for the Okayama models. It would be interesting nevertheless to examine how these 1F XFEL models may correlate with the known magnetic and spectroscopic properties of the S_2_ state. For this purpose, we assume explicitly the charge and spin multiplicity of the S_2_ state for the 1F models in the corresponding calculations in order to enable comparison against experimental data. The S_2_ state is the spectroscopically most well-characterized state of the OEC. It shows a multiline EPR signal centered at *g* ≈ 2,^127^ attributed to a low-spin-ground state of effective spin *S* = 1/2 that results from antiferromagnetic exchange interactions between the Mn spin centers. The multiline signal includes more than 18 resolved lines induced by the electron–nuclear hyperfine interaction of the four ^55^Mn nuclei (*I* = 5/2) with the electron spin. Determination of these ^55^Mn hyperfine interactions has been made possible by pulse electron nuclear double resonance (ENDOR) experiments.^18,19,41,51,54,55^ This ENDOR analysis has enabled a more thorough examination of the electronic structure of the S_2_ state by unambiguously suggesting that the cluster contains one Mn(III) ion and three Mn(IV) ions.

Τhe pairwise exchange coupling constants between the Mn spin centers of the 1F XFEL models were calculated with broken-symmetry DFT. Importantly, the spin populations obtained for the Okayama models do not correspond to one *S* = 2 and three *S* = 3/2 spin centers, but they rather indicate two *S* = 2 and two *S* = 3/2 spin centers in the cluster; thus, it is impossible to apply the same broken-symmetry analysis to those models. In other words, the Okayama 1F models are strictly incompatible with the S_2_-state magnetism and spectroscopy. The results for the **6W1P** and **6DHF** models are shown in Table S12. Based on the calculated exchange coupling constants, the complete spin ladder was obtained for each model from the diagonalization of the Heisenberg Hamiltonian. The computed ground spin states for **6W1P-A** and **6W1P-B** models are *S* = 11/2 and *S* = 13/2, respectively, which is inconsistent with experiment. The computed ground spin state for **6DHF-A** and **6DHF-B** models is *S* = 1/2 (Table S12), the predicted ground state spin configuration is α–β–β–α, and the energy differences between the two lowest states of the spin ladder are 45 and 59 cm^–1^ for monomers A and B, respectively, whereas the experimental estimates are 24–26 cm^–1^.^128^

Therefore, **6DHF** is the *only* existing 1F XFEL model that can in principle reproduce the magnetic properties of the S_2_ state, *if it is assigned* the electronic structure of the S_2_ state.

The calculated ^55^ Mn isotropic hyperfine coupling constants, *A*_iso_, for models **6DHF-A** and **6DHF-B** are given in Table 2 and are compared to the computed values for the QM model of the S_2_ state and to experimental values. The corresponding isotropic and anisotropic onsite hyperfine values for the Mn ions are given in Table S13. The calculated Mn2 and Mn3 ^55^Mn hyperfine coupling constants are consistent with experiment, but Mn1 and Mn4 show a stronger deviation, which reflects inaccurate description of their coordination geometry by the XFEL models. Overall, the **6DHF** cores produce a pattern of two large and two small ^55^Mn HFCs, in contrast to both QM-optimized models of the S_2_ state and with experimental data on the S_2_ state. Overestimation of the Mn1–N_His332_ bond lengths in the **6DHF** models is suggested by the calculated N_His332_ isotropic hyperfine coupling constants of 2.8 and 1.7 MHz for monomers A and B, respectively (Table S14), which are significantly smaller than the experimental value of 7.1 MHz. This type of inconsistency between ligand superhyperfine and metal OS^129^ is indicative of structural inaccuracies even for a site with uncontested OS.

**Table 2:** Computed Projected ^55^Mn Isotropic Hyperfine Coupling Constants (in MHz) for the 1F **6DHF** XFEL Models and for the QM Model of the S_2_ State, Compared to Experimentally Reported Values

	^55^Mn |*A*_iso_|			
**6DHF-A**	371 (Mn1)	303 (Mn4)	236 (Mn2)	213 (Mn3)
**6DHF-B**	335 (Mn1)	333 (Mn4)	237 (Mn2)	212 (Mn3)
QM, S_2_	277 (Mn4)	227 (Mn1)	214 (Mn2)	180 (Mn3)
Exp. S_2_ (ref ^130^)	307	209	204	190
Exp. S_2_ (ref ^130^)	310	242	205	194
Exp. S_2_ (ref ^51^)	333	230	227	194

In conclusion, the magnetic properties of the S_2_ state are impossible to reproduce by the Okayama 1F XFEL models, because Mn4 is clearly reduced to Mn(III) and the Mn OSs cannot be described as III–IV–IV–IV. Among the Berkeley models, the **6DHF** monomers **A** and **B** can reproduce the experimentally known ground and first excited spin states, although they are still not in sufficient agreement with ^55^Mn and ^14^N hyperfine coupling tensors.

### 3.7 Discussion of Results on the 0F and 1F Models

Modern SFX-XFEL crystallography of the OEC has led to unprecedented structural insights, reaching a level where structural models of distinct catalytic intermediates can be proposed. This fueled extensive debates and suggested revisions of long-held mechanistic scenarios, particularly after the publication of unexpected and controversial two-flash (2F) structural models that are supposed to depict the S_3_ state of the OEC. The results and analysis presented in this study show that the Mn OSs that correspond to the proposed 0F and 1F XFEL models are not consistent with the S_1_ and S_2_ states of the OEC.

After presenting the results of our analysis, which stand independently of experimental considerations, we turn our attention to a nonexhaustive list of proposed hypotheses that may serve as plausible explanations. We emphasize that these are conjectures and do not strictly follow from the present theoretical analysis of the XFEL models. First, the samples might be more reduced than the nominal S-state. This could be for example due to radiation-induced Mn reduction.^116,117^ Another possibility is that the samples might be asynchronized due to the conditions of dark adaptation and due to incomplete S-state transition,^70,94,131^ leading to a mixture of S_0_ and S_1_ states in 0F samples and a mixture of S_0_, S_1_, and S_2_ states in 1F samples. Additionally, the resulting structural models may be compromised by inaccurate quantification of S-state advancement/composition and by inadequate projection of the more reduced S-states from the diffraction data.^92,132^

In terms of the experimental approaches, it is noted that the Berkeley group confirmed with *in situ* X-ray emission spectroscopy (XES) that the XFEL beams do not affect the OEC under the experimental conditions used.^133,134^ In addition, they excluded from the analysis samples that XES had shown to contain Mn(II) and were therefore considered overreduced. Moreover, to quantify the S-state composition in the PSII microcrystals, the Okayama group used flash-induced Fourier transform infrared (FTIR) difference spectroscopy,^135^ whereas Berkeley investigators used XES performed simultaneously, and flash-induced oxygen evolution measurements (MIMS) for O_2_ detection performed before the XRD experiment. The efficiency of the S-state cycling was additionally quantified by flash-induced O_2_ yields and the oscillation of the multiline signal of the S_2_ state with a period of four as a function of the flash number, carried out by the Berkeley group. However, the aforementioned methods cannot distinguish between the two monomers; thus, asynchronized S-state progression, potentially caused by crystal packing, cannot be accounted for in data interpretation. A potentially relevant observation is the report by both groups of the disappearance of a water molecule in the proximity of O4 in the 1F and 2F states and reappearance in the 3F state. It is unclear what this observation means, particularly because no change on the protonation state of O4 is expected in the S_1_ → S_2_ transition (the bridge is unprotonated) and it does not directly relate to oxidation events. We can speculate, however, on a possible explanation for this observation in the context of S-state mixing. Specifically, if the 0F models reflect some of the S_0_-state features and if the S_0_ state has a protonated O4 bridge, then progression to S_1_ for this population would involve proton transfer to the water molecule that is no longer resolved in the 1F model.

Another possibility is that the XFEL models may—additionally—reflect an average of coexisting conformers within the *same* S-state.^49,61,136−138^ It is noted that the OEC exists in spectroscopically distinct forms in each S-state and the equilibrium between these forms is highly sensitive to environmental factors.^35,50^ For example, small perturbations in pH and temperature,^139,140^ ion concentration,^141−147^ cryoprotectants,^148^ point mutations,^149−152^ and protein depletion even at a long (>10 Å) distance from the OEC cluster^153−155^ are sufficient to shift the equilibrium between the low-spin (*g* ∼ 2) and high-spin (*g* ≥ 4.1) EPR signals of the S_2_ state, as well as between the *g* ∼ 4.8 and *g* ∼ 12 EPR signals of the S_1_ state.^156,157^ Given that the total effective spin state and spectroscopic signature arise from the magnetic exchange interactions among the Mn ions, which are in turn sensitive to the structure of the cluster, this spectroscopic heterogeneity likely originates from distinct conformations of the Mn_4_CaO_5_ cluster. The two different EPR signals observed in the S_1_ state have been attributed to orientational Jahn–Teller isomerism, where the pseudo-Jahn–Teller elongation axis of Mn4(III) can be either collinear or perpendicular to that of Mn1(III).^61^ However, this hypothesis does not justify the reduced Mn3(III) in the 0F XFEL models. Interestingly, the first 0F XFEL model, reported by Suga et al. in 2015,^72^ was also found to reflect a mixture of S_1_ and S_0_ states.^136^ Among the hypotheses proposed to explain the origin of distinct EPR signals in the S_2_ state, one scenario is that of valence isomerism, where valence exchange occurs between the terminal Mn1 and Mn4 ions, so that the Mn(III) ion is relocated from Mn1 (majority low-spin form) to Mn4 (minority high-spin form).^49^ Thus, it is not inconceivable that a small population of the S_2_ state with Mn4(III) may lead to apparent axial elongation at the Mn4 ion as observed in 1F XFEL models. Given that crystallography at the present level cannot resolve such heterogeneous populations, this might be manifested in our analysis as partial reduction of the Mn4 ion from its expected IV oxidation state. Another structure-based hypothesis that can explain the emergence of high-spin isoforms in the S_2_ state of the OEC is that of early binding of a water molecule (or hydroxy ligand) on Mn1.^158^ In the corresponding QM models presented by Pushkar and co-workers, the Mn(III) ion is relocated to the Mn2 or Mn3 ions. In the latter case, an admixture of such form could contribute to the observed axial elongation of the Mn3 ion in the 1F structures. Overall, however, given the limited reliability of even the best available crystallographic models, an attempt to identify possible isomers of the S_2_ state from 1F XFEL models seems misguided.

It is pertinent to reiterate at this point that our analysis has not explicitly incorporated the issue of limited experimental resolution, which is not obvious how to achieve from the side of quantum chemical modeling. However, the reported models have structural characteristics that systematically reflect *specific* Mn OSs in the *same* Mn ions. Nevertheless, our methods of analysis extend beyond the empirical criterion of axial distortion to more sophisticated methods such as the EOS analysis and calculation of magnetic properties, which are not based on specific bond lengths, but on the overall electron (or spin) density distribution in the cluster. Using those methods, clear patterns emerge concerning the differences between the two groups data; i.e., the Okayama models *appear* to be systematically more reduced, whereas Berkeley models have larger differences among the two monomers and *appear* less reduced than corresponding Okayama models (always assuming the high-oxidation paradigm as reference). Moreover, no obvious correlation can be established between higher resolution of XFEL models and their OS definition according to the available data. Consequently, the observed deviations from the expected Mn OSs cannot be attributed *exclusively* to resolution limitations.

Finally, prompted by the comments of one reviewer of this work, we note that the evaluation of the XFEL models in our study is performed on the basis of the Mn OSs supported by the high OSs paradigm (HOP), which is supported by multiple lines of evidence.^16,18,19,33,34,52,159,160^ An alternative hypothesis is that the Mn OSs follow the low OSs paradigm (LOP), where each state has two more unpaired electrons than the corresponding state of the HOP. Therefore, in the S_1_ state the Mn OSs are Mn(III)_4_ or Mn(II)Mn(III)_2_Mn(IV) instead of Mn(III)_2_Mn(IV)_2_; in the S_2_ state they are Mn(III)_3_Mn(IV) or Mn(II) Mn(III)Mn(IV)_2_ instead of Mn(III)Mn(IV)_3_; in the S_3_ state they are Mn(III)_2_Mn(IV)_2_ instead of Mn(IV)_4_; and in the S_0_ state they are Mn(II)Mn(III)_3_ instead of Mn(III)_3_Mn(IV).^161−167^ Among the 0F models, only the Okayama models are consistent with Mn(II)Mn(III)_2_Mn(IV), in line with expectations according to the LOP. However, the corresponding 1F models are also Mn(II)Mn(III)_2_Mn(IV), which implies incomplete S-state progression. The Berkeley models are not consistent with the LOP because they already appear more oxidized than the expectations of this paradigm. The practice by the Berkeley group of rejecting Mn(II)-containing samples may be one reason that the mean oxidation state of the OEC in their models is pushed upward relative to the data of the Okayama group. In conclusion, our results do not support that the XFEL models can be considered consistent with the LOP in either the 0F and 1F state.

In conclusion, our analysis shows that the Mn–O distances of the XFEL models do not correspond to those expected for the respective Mn OSs. While these features may have a physical basis, such as limitations inherent in the experimental protocols, the intricate nature of the OEC S-state transitions, and in-state isomerism, they may also reflect limitations in data interpretation and resolution.

### 3.8 The S_3_ State and Implications for Structure-Based Mechanistic Inferences

In light of the above conclusions regarding the 0F and 1F models, it is expected that extracting meaningful information for the Mn OSs from the 2F models would be even more challenging. Since the 2F samples are derived from one-flash illumination of the 1F samples, the 2F models are likely to also reflect mixtures of S-states. In addition, the S_3_ state is known to be intrinsically heterogeneous, with its nature and composition being a continuing subject of debate,^20−24,26,88,89,168−173^ along with the associated complex S_2_ → S_3_ transition.^21,82,140,168,174−183^ Therefore, the XFEL models would also be affected by in-state structural heterogeneity.

**Figure 7:**
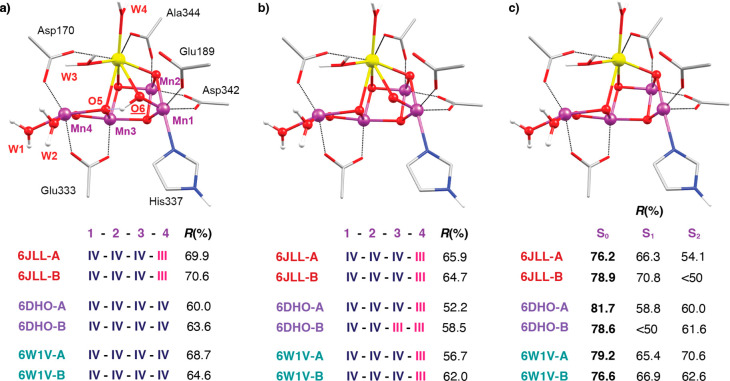
Inorganic core of the OEC from the two-flash **6DHO-A** XFEL model with (a) protonated O6, (b) deprotonated O6, and (c) the O6 atom omitted. *R*(%) values for the EOS are reported for combinations of charge and multiplicity that correspond to the S_3_ state (a and b) and to the S_0_–S_2_ states (c).

Despite these complications, for the sake of completeness we carried out BVS and EOS analysis for the latest 2F XFEL models. In Figure S6, the Mn OSs derived from BVS analysis for the 2F structures **6JLL**, **6W1V**, and **6DHO** are plotted. As in the 0F and 1F models, the geometry of the Mn3 and Mn4 ions corresponds to Mn(III), except for the **6JLL** Okayama models, where the Mn4 corresponds best to Mn(II). The EOS analysis results are presented in Figure 7 as well as Tables S15 and S16. Imposing the charge and multiplicity of the S_3_ state on 2F structural models does not lead to Mn OSs of IV–IV–IV–IV at all unless the O6 ligand is protonated. In that case, the **6DHO** and **6W1V** models do reproduce the S_3_ state OSs. Alternative interpretations of the 2F electron densities have been proposed,^92^ where the O6 ligand is absent. Therefore, we also performed EOS analysis with the 2F models without the O6 ligand, assuming the charge and multiplicity of the S_0_, S_1_, and S_2_ states. This indeed yields higher *R*(%) values and, remarkably, using the charge and multiplicity of the S_0_ state leads to the highest *R*(%) values for the 2F structures. Overall, however, the values are too low to allow tracing any meaningful correspondence between the 2F structures and any “pure” combination of Mn OSs, or indeed of any S-state mixture.

Overall, the “best case” among all SFX-XFEL models can be considered to be the structures of Kern et al.^79^ Our results, in agreement with the work of Amin,^94^ suggest that if any of the existing XFEL models can be viewed as an adequate approximation of a state *higher* than the dark-stable S_1_, this can only be the 1F models **6DHE** and **6DHF**, even though their Mn OSs derived from our analysis are still not fully consistent with spectroscopy. It is important to note that the corresponding 2F structure of the same series, **6DHO**,^79^ is the only XFEL model hypothetically representative of the S_3_ state in which the distance between O5 and the Mn1 coordinated oxygen atom (denoted as O6 or Ox) inserted during the S_2_ → S_3_ transition is too long (2.1 Å) to allow for an interpretation of peroxide or oxyl-oxo early onset O–O bond formation after two flashes. If this is the case, then it suggests that a large part of recent literature that discusses early onset O–O bond formation in the S_3_ state is motivated by an incorrect literal interpretation of crystallographic models.

## 4 Conclusions

Serial femtosecond crystallography with X-ray free electron lasers allowed the extraction of structural information for states of the OEC cycle beyond the dark-stable S_1_ state. However, key structural parameters of the 1F models raise ambiguities concerning the reflected Mn oxidation states. We employed a series of criteria ranging from purely structure-based metrics to quantum chemistry-based analysis of charge and spin density distribution, and finally to magnetic and spectroscopic properties calculations, in order to examine whether the latest SFX-XFEL 1F models of the OEC are in fact representative of the S_2_ state. The metrics used in the present work are not restricted to a discretized analysis that leads to formal integer oxidation states but allow for a quantification of goodness-of-fit and physical oxidation state assignments. Our results show that already the 0F SFX-XFEL models (models of the resting S_1_ state) are problematic because Mn3 appears largely reduced to Mn(III), a feature more consistent with the III–IV–III–III Mn OSs of the S_0_ state.

All Okayama 0F and 1F models considered here seem over-reduced, but monomers A and B appear synchronized. The similarity among all the Okayama structures suggests that the observed Mn OSs are not caused by experimental uncertainty of the Mn–O bond lengths, but rather reflect a systematic elongation. In the Berkeley 0F and 1F XFEL models **6W1O** and **6W1P**, the monomers A and B are not synchronized in the same S-state and appear reduced to a smaller extent. The 0F model that proved to be most consistent with the Mn OSs of the S_1_ state (**6DHE**) also gives the best 1F model of the S_2_ state (**6DHF**). In the 1F model **6DHF**, Mn4 appears somewhat reduced but to a much smaller degree than in any other 1F model, and the monomers A and B are synchronized. This structural model is also the most consistent with the experimental data on the S_2_ state derived from spectroscopy and with the known properties of this state expected from the catalytic progression of the OEC. The geometry of this structure is closer to the QM model of the S_2_ state than to the QM model of the S_1_ state, and the computed exchange coupling constants are consistent with an *S* = 1/2 ground state. Even though the calculated ^55^Mn and ^14^N experimental hyperfine coupling tensors for this model deviate from experiment compared to those of the QM model of the S_2_ state, they can be considered a satisfactory approximation, albeit with great room for improvement. The issues identified for 0F and 1F XFEL models suggest a high degree of unreliability for the 2F models. Our results show that all problems are indeed compounded for 2F models, showing that the latter cannot be reliably interpreted in terms of geometric/electronic structural forms of the actual S_3_ state.

In conclusion, the present results highlight and quantify limitations in state-specific interpretations of current SFX-XFEL models of the OEC. The existing structural models derived from such studies have not yet achieved the specificity required to provide reliable atomic-level descriptions of catalytic intermediates at the level required for comparisons with (or utilization by) electronic structure investigations. Nevertheless, our work suggests measures that can be used to evaluate S-state specificity of such structural models in an intrinsic way. We anticipate that incorporation of insights provided by the established combination of quantum chemistry and spectroscopy into the development and refinement of crystallographic models will eventually focus efforts toward correctly evaluating the information content of crystallography and enable us to move more confidently toward uncovering the remaining secrets of biological water oxidation.

## Supporting Information Available

Additional methodological details; key bond lengths of XFEL models for B monomers (Figure S1); RMSD of the inorganic core of 1F XFEL models from QM models of the S_1_ and S_2_ states (Figure S2); axial elongation descriptors for Mn ions of all XFEL models (Table S1); bond valence sum Mn OSs for 0F and 1F XFEL models and for QM models of the S_1_ and S_2_ states (Table S2); bond valence sum Mn OSs for other reported QM models of the S_2_ state (Table S3); detailed bond valence sum values correlated with Mn OSs (Table S4); averaged axial and equatorial bond lengths for Mn ions of 0F and 1F XFEL structures (Table S5); bond valence sum Mn OSs using the averaged axial and equatorial bond lengths (Table S6); Mn OSs for 0F XFEL structures derived from BVS analysis using specific ion parameters for specific Mn centers (Figure S3); Mulliken spin populations (Table S7); QTAIM spin populations (Table S8, Figure S4); calculated Mn OSs and corresponding *R*(%) values derived from EOS analysis of dinuclear Mn complexes (Figure S5); *R*(%) values for EOS calculated using combinations of charge and multiplicity that correspond to different S-states for the 0F and 1F XFEL structures (Table S9); occupation numbers of frontier EFOs (Table S10); *R*(%) values for other reported QM models of the S_2_-state (Table S11); calculated exchange coupling constants and spin state properties (Table S12); spin projection factors and ^55^Mn hyperfine coupling constants (Table S13); ^14^N hyperfine coupling constants for the N(His332) ligand (Table S14); BVS Mn OSs for 2F XFEL structures (Figure S6); and *R*(%) values and EOS assignments for 2F XFEL structures (Tables S15, S16). (PDF)
Cartesian coordinates of all structural models discussed in this work. (TXT)
