# Supplementary material for: Does Serial Femtosecond Crystallography Depict State-Specific Catalytic Intermediates of the Oxygen-Evolving Complex?
Source: J Am Chem Soc. 2023 May 3;145(19):10604–21. doi: 10.1021/jacs.3c00489 (PMC10197136; doi:10.1021/jacs.3c00489)
Supplement: Supplementary file 1 — ja3c00489_si_001.pdf [file ja3c00489_si_001.pdf]

Supporting Information

Does Serial Femtosecond Crystallography Depict State-Specific Catalytic Intermediates of the Oxygen-Evolving Complex?

Maria Drosou,[a] Gerard Comas-Vilà,[b] Frank Neese,[a] Pedro Salvador,[b]* Dimitrios A. Pantazis[a]*

[a] Max-Planck-Institut für Kohlenforschung Kaiser-Wilhelm-Platz 1, 45470, Mülheim an der Ruhr, Germany

[b] Institute of Computational Chemistry and Catalysis, Chemistry Department, University of Girona, Montilivi Campus, Girona, Catalonia 17003, Spain

E-mail: pedro.salvador@udg.edu
E-mail: dimitrios.pantazis@kofo.mpg.de

Additional Methodological Details

The coordinates of the OEC models were extracted from the crystallographic coordinates (pdb files) of each XFEL structure and, subsequently, H atoms were added manually. Geometry optimization was performed only for the hydrogen atoms using the charge and multiplicity of the S_1, S_2, and S_3 states for the 0F, 1F, and 2F models, respectively. Hydrogen optimizations were performed with W2 in its aquo form, and in the high-spin state, therefore the imposed charge and multiplicity were (1, 15) for 0F models, (2, 14) for 1F models and (2, 13) for 2F models. For the EOS analysis of the XFEL structures in the S_0 state, additional hydrogen optimizations were performed with protonated O5 or O4 using charge and multiplicity (1, 16).

Single-point calculations on each model using multiple combinations of charge and multiplicity corresponding to different S-states were then performed. Different protonation states of W2 (H_2O or OH) and O6 (OH or O) were examined. For the calculation of the pairwise Mn-Mn exchange coupling constants for the 1F models using the S_2 state charge and multiplicity (i.e. 14 for the high-spin solution), single-point calculations of different spin configurations, i.e. *α-α-α-β*, *α-α-β-α*, *α-β-α-α*, *β-α-α-α*, *α-α-β-β*, *α-β-α-β*, and *α-β-β-α*, were carried out. The resulting overdetermined system of equations was solved by singular value decomposition to yield the pairwise exchange coupling constants, J_{ij}. The complete energy ladder of spin eigenstates was calculated through diagonalization of the Heisenberg Hamiltonian:

$$\hat{H} = -2 \sum_{i<j} J_{ij}\, \hat{\boldsymbol{S}}_i \hat{\boldsymbol{S}}_j$$

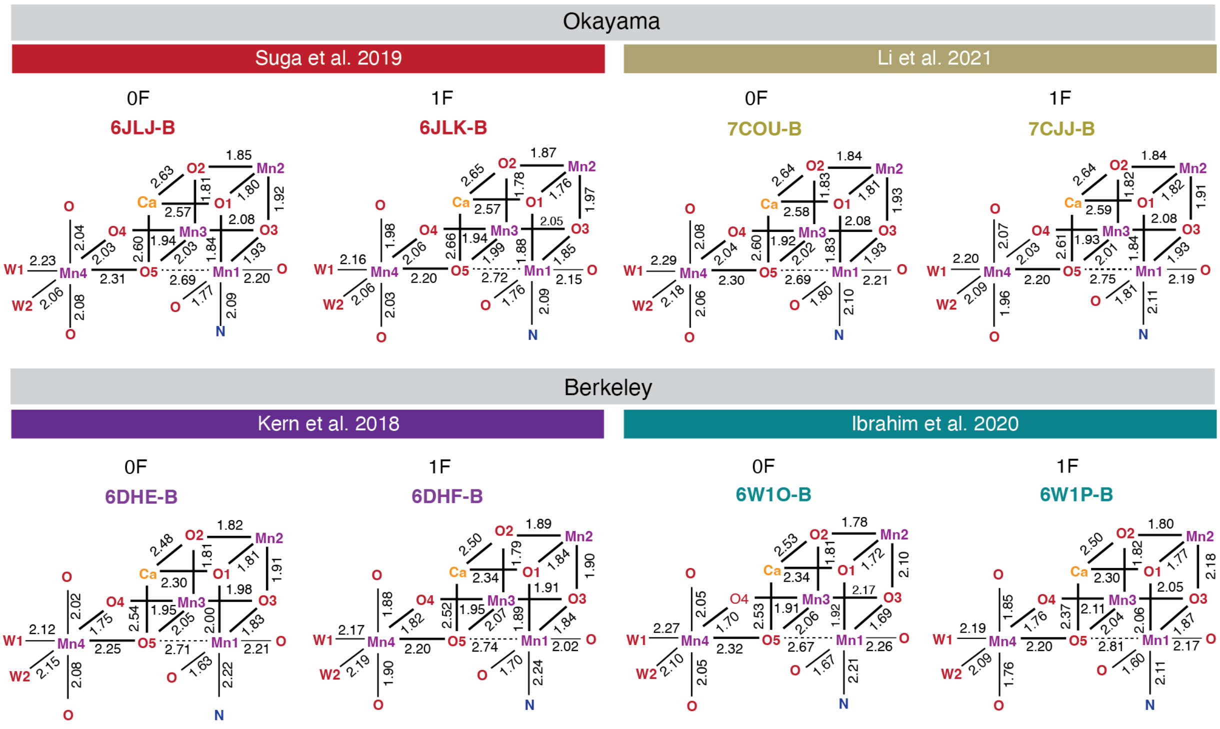

Figure S1. Key bond lengths of XFEL models (monomer B) of the S_1 and S_2 states; Okayama models include the **6JLJ** (0F) and **6JLK** (1F) reported by Suga et al.[1] in 2019, and the **7COU** (0F) and **7CJJ** (1F) reported by Li et al.[2] in 2021. Berkeley models include the **6DHE** (0F) and **6DHF** (1F) reported by Kern et al. in 2018,[3] and **6W1O** (0F) and **6W1P** (1F) reported by Ibrahim et al.[4] in 2020.

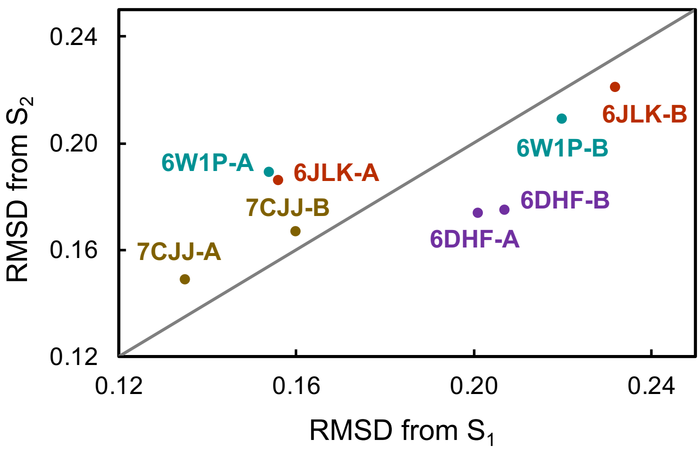

Figure S2. RMSD of the inorganic core (Mn_4CaO_5) of 1F XFEL models from QM models of the S_1 and S_2 states.

Table S1. Axial elongation descriptors r_1–r$_3$ and r_1–r$_2$ for Mn ions of all XFEL models.

0F		Okayama				Berkeley			
		6JLJ		7COU		6W1O		6DHE	
		A	B	A	B	A	B	A	B
Mn1	r_1–r$_3$	0.335	0.339	0.320	0.333	0.326	0.448	0.343	0.418
	r_1–r$_2$	0.279	0.272	0.261	0.274	0.214	0.225	0.192	0.203
Mn2	r_1–r$_3$	0.059	0.064	0.026	0.024	0.155	0.049	0.049	0.055
	r_1–r$_2$	0.025	0.035	0.001	0.012	0.047	0.022	0.016	0.015
Mn3	r_1–r$_3$	0.075	0.081	0.059	0.071	0.107	0.068	0.089	0.061
	r_1–r$_2$	0.041	0.035	0.024	0.040	0.057	0.018	0.060	0.020
Mn4	r_1–r$_3$	0.128	0.130	0.135	0.128	0.063	0.226	0.137	0.134
	r_1–r$_2$	0.125	0.120	0.133	0.105	0.035	0.142	0.081	0.074

1F		Okayama				Berkeley			
		6JLK		7CJJ		6W1P		6DHF	
		A	B	A	B	A	B	A	B
Mn1	r_1–r$_3$	0.331	0.359	0.322	0.342	0.298	0.431	0.295	0.346
	r_1–r$_2$	0.279	0.257	0.271	0.285	0.152	0.231	0.172	0.177
Mn2	r_1–r$_3$	0.033	0.050	0.038	0.038	0.029	0.104	0.020	0.043
	r_1–r$_2$	0.021	0.046	0.013	0.010	0.006	0.089	0.002	0.024
Mn3	r_1–r$_3$	0.060	0.049	0.065	0.066	0.064	0.090	0.060	0.033
	r_1–r$_2$	0.055	0.015	0.025	0.026	0.038	0.077	0.020	0.012
Mn4	r_1–r$_3$	0.143	0.102	0.136	0.105	0.175	0.221	0.104	0.169
	r_1–r$_2$	0.079	0.070	0.096	0.081	0.146	0.153	0.104	0.104

Table S2. Bond valence sum Mn OSs for all 0F and 1F XFEL models and for the QM models of the S_1 and S_2 states.

0F	Okayama				Berkeley				
	6JLJ		7COU		6W1O		6DHE		QM
	A	B	A	B	A	B	A	B	S_1
Mn1	3.389	3.406	3.105	3.245	2.649	4.658	3.489	4.113	2.886
Mn2	3.792	3.733	3.559	3.617	4.506	4.038	4.292	3.988	3.836
Mn3	3.111	3.160	3.180	3.165	4.248	3.076	3.645	3.605	3.784
Mn4	1.929	1.917	1.945	2.098	3.513	3.068	3.018	2.867	2.939

1F	Okayama				Berkeley				
	6JLK		7CJJ		6W1P		6DHF		QM
	A	B	A	B	A	B	A	B	S_2
Mn1	3.191	3.584	3.098	3.202	3.859	4.262	3.659	3.965	2.929
Mn2	3.752	3.756	3.621	3.683	4.717	3.688	4.290	4.000	3.819
Mn3	3.114	3.533	3.228	3.234	4.514	3.402	3.759	3.562	3.885
Mn4	2.541	2.516	2.499	2.467	3.274	3.725	3.396	3.152	3.738

Table S3. Bond valence sum Mn OSs for four reported QM models of the S_2 state.

	**Ref. 5**	**Ref. 6**	**Ref. 7**	**Ref. 8**
Mn1	2.929	3.126	3.184	3.137
Mn2	3.819	4.087	4.055	4.019
Mn3	3.885	4.022	4.032	4.138
Mn4	3.738	3.934	3.923	3.889

Table S4. Bond valence sum values calculated using equation (1) in the main text, for a range of bond distances (across each row) and for a range of axial distortion degrees, as quantified by the ratio of the averaged axial Mn–O bond lengths to the averaged equatorial Mn–O bond lengths. Each color represents the predicted Mn oxidation state, i.e. green for Mn(IV), yellow for Mn(III) and orange for Mn(II). The first table (**a**) is computed using the parameters for Mn(III), $R_0 = 1.823$ Å and $B = 0.247$ Å and the second table (**b**) is computed using the parameters for Mn(IV) ions $R_0 = 1.750$ Å and $B = 0.374$ Å.

(a)

	$\sum_{i=1}^{6} R_i$																
A/*E*	**11.4**	**11.5**	**11.6**	**11.7**	**11.8**	**11.9**	**12**	**12.1**	**12.2**	**12.3**	**12.4**	**12.5**	**12.6**	**12.7**	**12.8**	**12.9**	**13**
**0.70**	9.34	8.83	8.36	7.91	7.48	7.08	6.70	6.34	6.00	5.68	5.38	5.09	4.82	4.56	4.32	4.09	3.87
**0.72**	8.46	7.99	7.55	7.14	6.74	6.37	6.02	5.69	5.38	5.08	4.81	4.54	4.29	4.06	3.84	3.63	3.43
**0.74**	7.72	7.28	6.87	6.48	6.12	5.77	5.45	5.14	4.85	4.58	4.32	4.08	3.85	3.64	3.43	3.24	3.06
**0.76**	7.08	6.67	6.29	5.92	5.58	5.26	4.96	4.68	4.41	4.15	3.92	3.69	3.48	3.28	3.09	2.92	2.75
**0.78**	6.54	6.16	5.79	5.45	5.13	4.83	4.55	4.28	4.03	3.79	3.57	3.36	3.17	2.98	2.81	2.65	2.49
**0.80**	6.08	5.72	5.38	5.06	4.75	4.47	4.20	3.95	3.71	3.49	3.28	3.09	2.91	2.73	2.57	2.42	2.27
**0.82**	5.70	5.35	5.03	4.72	4.43	4.16	3.91	3.67	3.45	3.24	3.04	2.86	2.69	2.52	2.37	2.23	2.09
**0.84**	5.38	5.05	4.74	4.44	4.17	3.91	3.67	3.44	3.23	3.03	2.85	2.67	2.51	2.35	2.21	2.07	1.94
**0.86**	5.12	4.80	4.50	4.22	3.95	3.70	3.47	3.26	3.05	2.86	2.68	2.51	2.36	2.21	2.07	1.94	1.82
**0.88**	4.91	4.59	4.30	4.03	3.78	3.54	3.31	3.10	2.91	2.72	2.55	2.39	2.24	2.10	1.96	1.84	1.72
**0.90**	4.74	4.43	4.15	3.88	3.64	3.40	3.19	2.98	2.79	2.61	2.45	2.29	2.14	2.01	1.88	1.76	1.65
**0.92**	4.60	4.31	4.03	3.77	3.53	3.30	3.09	2.89	2.70	2.53	2.37	2.21	2.07	1.94	1.81	1.70	1.59
**0.94**	4.51	4.22	3.94	3.69	3.45	3.22	3.02	2.82	2.64	2.47	2.31	2.16	2.02	1.89	1.76	1.65	1.54
**0.96**	4.44	4.15	3.88	3.63	3.39	3.17	2.97	2.77	2.59	2.42	2.27	2.12	1.98	1.85	1.73	1.62	1.51
**0.98**	4.40	4.12	3.85	3.60	3.36	3.14	2.94	2.75	2.57	2.40	2.24	2.10	1.96	1.83	1.71	1.60	1.50
**1.00**	4.39	4.11	3.84	3.59	3.35	3.14	2.93	2.74	2.56	2.39	2.24	2.09	1.95	1.83	1.71	1.60	1.49
**1.02**	4.40	4.12	3.85	3.60	3.36	3.14	2.94	2.75	2.57	2.40	2.24	2.10	1.96	1.83	1.71	1.60	1.50
**1.04**	4.44	4.15	3.88	3.63	3.39	3.17	2.96	2.77	2.59	2.42	2.26	2.12	1.98	1.85	1.73	1.62	1.51
**1.06**	4.49	4.20	3.92	3.67	3.43	3.21	3.00	2.81	2.62	2.45	2.29	2.15	2.01	1.88	1.75	1.64	1.53
**1.08**	4.56	4.26	3.99	3.73	3.49	3.26	3.05	2.86	2.67	2.50	2.34	2.19	2.04	1.91	1.79	1.67	1.56
**1.10**	4.64	4.35	4.07	3.80	3.56	3.33	3.12	2.92	2.73	2.55	2.39	2.23	2.09	1.96	1.83	1.71	1.60
**1.12**	4.75	4.44	4.16	3.89	3.64	3.41	3.19	2.99	2.80	2.62	2.45	2.29	2.15	2.01	1.88	1.76	1.65
**1.14**	4.86	4.55	4.26	3.99	3.74	3.50	3.28	3.07	2.87	2.69	2.52	2.36	2.21	2.07	1.94	1.81	1.70
**1.16**	5.00	4.68	4.38	4.11	3.85	3.60	3.37	3.16	2.96	2.77	2.60	2.43	2.28	2.14	2.00	1.87	1.76
**1.18**	5.14	4.82	4.51	4.23	3.96	3.71	3.48	3.26	3.06	2.86	2.68	2.52	2.36	2.21	2.07	1.94	1.82
**1.20**	5.30	4.97	4.65	4.36	4.09	3.84	3.60	3.37	3.16	2.96	2.78	2.60	2.44	2.29	2.15	2.01	1.89
**1.22**	5.47	5.13	4.81	4.51	4.23	3.97	3.72	3.49	3.27	3.07	2.88	2.70	2.53	2.38	2.23	2.09	1.96
**1.24**	5.65	5.30	4.97	4.66	4.38	4.11	3.85	3.62	3.39	3.18	2.99	2.80	2.63	2.47	2.32	2.17	2.04
**1.26**	5.84	5.48	5.14	4.83	4.53	4.26	4.00	3.75	3.52	3.31	3.10	2.91	2.74	2.57	2.41	2.26	2.13
**1.28**	6.04	5.67	5.33	5.00	4.70	4.41	4.14	3.89	3.66	3.43	3.23	3.03	2.85	2.67	2.51	2.36	2.22
**1.30**	6.26	5.88	5.52	5.19	4.87	4.58	4.30	4.04	3.80	3.57	3.35	3.15	2.96	2.78	2.62	2.46	2.31

(b)

	$\sum_{i=1}^{6} R_i$																
A/*E*	**11.4**	**11.5**	**11.6**	**11.7**	**11.8**	**11.9**	**12**	**12.1**	**12.2**	**12.3**	**12.4**	**12.5**	**12.6**	**12.7**	**12.8**	**12.9**	**13**
**0.70**	5.66	5.45	5.24	4.85	4.67	4.50	4.33	4.16	4.01	3.86	3.71	3.57	3.44	3.31	3.19	3.07	3.07
**0.72**	5.40	5.19	4.99	4.61	4.44	4.26	4.10	3.94	3.79	3.65	3.51	3.37	3.24	3.12	3.00	2.89	2.89
**0.74**	5.17	4.96	4.77	4.40	4.23	4.06	3.90	3.75	3.60	3.46	3.33	3.20	3.07	2.95	2.84	2.73	2.73
**0.76**	4.96	4.76	4.57	4.22	4.05	3.89	3.73	3.58	3.44	3.30	3.17	3.05	2.93	2.81	2.70	2.59	2.59
**0.78**	4.78	4.59	4.40	4.05	3.89	3.73	3.58	3.44	3.30	3.16	3.04	2.91	2.80	2.68	2.58	2.47	2.47
**0.80**	4.63	4.44	4.26	3.91	3.75	3.60	3.45	3.31	3.17	3.04	2.92	2.80	2.69	2.58	2.47	2.37	2.37
**0.82**	4.50	4.31	4.13	3.79	3.64	3.49	3.34	3.20	3.07	2.94	2.82	2.70	2.59	2.48	2.38	2.28	2.28
**0.84**	4.39	4.20	4.02	3.69	3.54	3.39	3.25	3.11	2.98	2.85	2.74	2.62	2.51	2.41	2.30	2.21	2.21
**0.86**	4.29	4.11	3.93	3.61	3.45	3.31	3.17	3.03	2.91	2.78	2.66	2.55	2.44	2.34	2.24	2.15	2.15
**0.88**	4.21	4.03	3.86	3.54	3.39	3.24	3.10	2.97	2.84	2.72	2.61	2.49	2.39	2.29	2.19	2.10	2.10
**0.90**	4.15	3.97	3.80	3.48	3.33	3.19	3.05	2.92	2.79	2.67	2.56	2.45	2.34	2.24	2.15	2.05	2.05
**0.92**	4.10	3.92	3.75	3.44	3.29	3.14	3.01	2.88	2.75	2.64	2.52	2.41	2.31	2.21	2.11	2.02	2.02
**0.94**	4.06	3.89	3.72	3.40	3.25	3.11	2.98	2.85	2.73	2.61	2.49	2.39	2.28	2.18	2.09	2.00	2.00
**0.96**	4.04	3.86	3.69	3.38	3.23	3.09	2.96	2.83	2.71	2.59	2.48	2.37	2.26	2.17	2.07	1.98	1.98
**0.98**	4.02	3.85	3.68	3.37	3.22	3.08	2.94	2.82	2.69	2.58	2.46	2.36	2.25	2.16	2.06	1.97	1.97
**1.00**	4.02	3.84	3.68	3.36	3.22	3.08	2.94	2.81	2.69	2.57	2.46	2.35	2.25	2.15	2.06	1.97	1.97
**1.02**	4.02	3.85	3.68	3.37	3.22	3.08	2.94	2.82	2.69	2.58	2.46	2.36	2.25	2.16	2.06	1.97	1.97
**1.04**	4.04	3.86	3.69	3.38	3.23	3.09	2.96	2.83	2.70	2.59	2.47	2.37	2.26	2.16	2.07	1.98	1.98
**1.06**	4.06	3.88	3.71	3.40	3.25	3.11	2.97	2.84	2.72	2.60	2.49	2.38	2.28	2.18	2.08	1.99	1.99
**1.08**	4.08	3.91	3.74	3.42	3.27	3.13	3.00	2.87	2.74	2.62	2.51	2.40	2.30	2.20	2.10	2.01	2.01
**1.10**	4.12	3.94	3.77	3.45	3.30	3.16	3.03	2.89	2.77	2.65	2.54	2.43	2.32	2.22	2.13	2.03	2.03
**1.12**	4.16	3.98	3.81	3.49	3.34	3.20	3.06	2.93	2.80	2.68	2.57	2.46	2.35	2.25	2.15	2.06	2.06
**1.14**	4.21	4.03	3.86	3.53	3.38	3.24	3.10	2.97	2.84	2.72	2.60	2.49	2.38	2.28	2.18	2.09	2.09
**1.16**	4.26	4.08	3.91	3.58	3.43	3.28	3.14	3.01	2.88	2.76	2.64	2.53	2.42	2.32	2.22	2.13	2.13
**1.18**	4.32	4.14	3.97	3.64	3.48	3.33	3.19	3.06	2.93	2.80	2.69	2.57	2.46	2.36	2.26	2.16	2.16
**1.20**	4.39	4.20	4.03	3.69	3.54	3.39	3.25	3.11	2.98	2.85	2.73	2.62	2.51	2.40	2.30	2.20	2.20
**1.22**	4.46	4.27	4.09	3.76	3.60	3.45	3.30	3.16	3.03	2.91	2.78	2.67	2.56	2.45	2.35	2.25	2.25
**1.24**	4.53	4.34	4.16	3.82	3.66	3.51	3.36	3.22	3.09	2.96	2.84	2.72	2.61	2.50	2.39	2.29	2.29
**1.26**	4.61	4.42	4.24	3.89	3.73	3.58	3.43	3.29	3.15	3.02	2.90	2.78	2.66	2.55	2.45	2.34	2.34
**1.28**	4.70	4.50	4.32	3.97	3.80	3.65	3.50	3.35	3.22	3.08	2.96	2.83	2.72	2.61	2.50	2.40	2.40
**1.30**	4.78	4.59	4.40	4.05	3.88	3.72	3.57	3.42	3.28	3.15	3.02	2.90	2.78	2.67	2.56	2.45	2.45

Table S5. Averaged axial and equatorial bond lengths for the Mn ions of the 0F and 1F XFEL structures.

0F		Okayama				Berkeley			
		6JLJ		7COU		6W1O		6DHE	
		A	B	A	B	A	B	A	B
Mn1	*A*	2.444	2.443	2.438	2.449	2.466	2.463	2.398	2.461
	E	1.907	1.908	1.929	1.918	1.993	1.874	1.931	1.918
Mn2	*A*	1.993	2.007	1.974	1.978	1.921	1.931	1.916	1.954
	E	1.920	1.920	1.954	1.946	1.868	1.928	1.859	1.893
Mn3	*A*	2.006	2.011	2.005	1.997	1.805	2.039	1.874	1.898
	E	2.011	2.002	1.995	2.005	1.942	2.011	1.977	1.988
Mn4	*A*	2.271	2.272	2.280	2.293	2.069	2.297	2.167	2.184
	E	2.050	2.052	2.046	2.089	1.983	1.975	1.976	2.002

1F		Okayama				Berkeley			
		6JLK		7CJJ		6W1P		6DHF	
		A	B	A	B	A	B	A	B
Mn1	*A*	2.457	2.436	2.453	2.470	2.360	2.490	2.343	2.378
	E	1.923	1.897	1.934	1.922	1.966	1.910	1.935	1.920
Mn2	*A*	1.978	2.004	1.987	1.978	1.864	1.913	1.856	1.871
	E	1.931	1.920	1.942	1.936	1.848	1.979	1.890	1.926
Mn3	*A*	1.978	1.997	2.002	2.002	1.860	2.080	1.874	1.931
	E	2.022	1.941	1.990	1.990	1.870	1.934	1.961	1.977
Mn4	*A*	2.214	2.183	2.227	2.201	2.201	2.193	2.114	2.185
	E	2.021	2.033	2.024	2.038	1.920	1.865	1.932	1.946

Table S6. Bond valence sum Mn OSs derived from equation 1 using the averaged axial and equatorial bond lengths.

0F	Okayama				Berkeley			
	6JLJ		7COU		6W1O		6DHE	
	A	B	A	B	A	B	A	B
Mn1	3.009	2.994	2.766	2.885	2.157	3.405	2.783	2.878
Mn2	3.586	3.541	3.416	3.453	4.183	3.720	4.274	3.887
Mn3	2.998	3.036	3.088	3.056	4.122	2.912	3.618	3.464
Mn4	2.291	2.278	2.300	2.083	2.997	2.658	2.843	2.666

1F	Okayama				Berkeley			
	6JLK		7CJJ		6W1P		6DHF	
	A	B	A	B	A	B	A	B
Mn1	2.817	3.132	2.706	2.825	2.467	2.947	2.787	2.911
Mn2	3.556	3.556	3.457	3.520	4.548	3.462	4.260	3.949
Mn3	3.021	3.436	3.124	3.126	4.392	3.274	3.714	3.414
Mn4	2.518	2.507	2.481	2.451	3.141	3.551	3.213	2.995

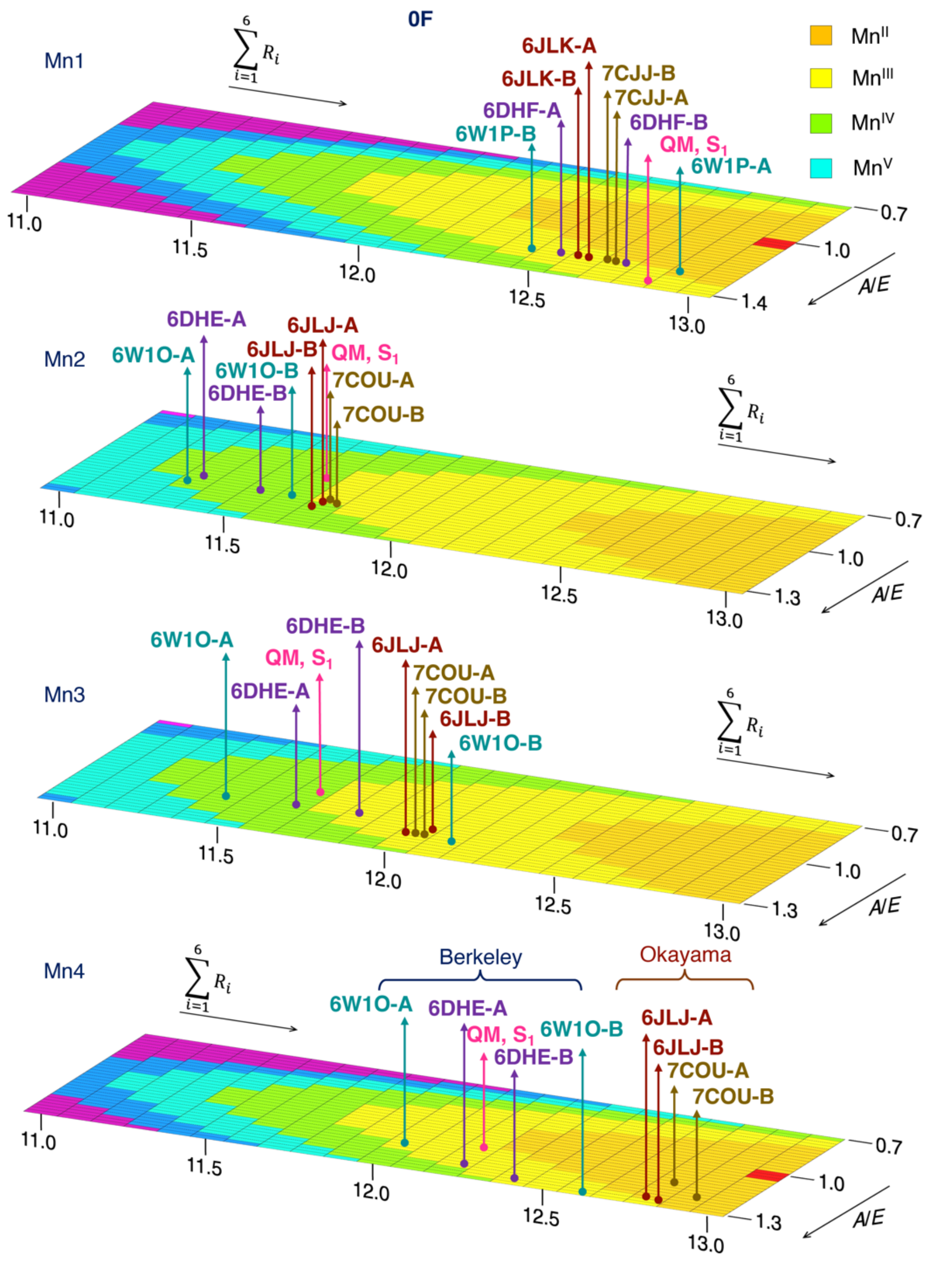

Figure S3. Mn OSs for the 0F XFEL structures derived from bond valence sum analysis using the parameters optimized for Mn(III) on Mn1 and Mn4 and the parameters optimized for Mn(IV) for Mn2 and Mn3 ions.

Table S7. Calculated Mulliken Mn spin populations for the 0F XFEL models assuming the total charge and spin multiplicity of the S_1 state and for the 1F XFEL models assuming the charge and multiplicity of the S_2 state.

0F	Okayama				Berkeley			
	6JLJ		7COU		6W1O		6DHE	
	A	B	A	B	A	B	A	B
Mn1	3.89	3.90	3.92	3.90	3.99	3.79	3.91	3.84
Mn2	2.93	2.94	3.00	2.96	2.88	2.84	2.93	2.94
Mn3	3.16	3.14	3.13	3.13	2.83	3.22	3.06	3.13
Mn4	4.07	4.08	4.06	4.12	3.82	3.88	3.86	3.90

1F	Okayama				Berkeley			
	6JLK		7CJJ		6W1P		6DHF	
	A	B	A	B	A	B	A	B
Mn1	3.86	3.81	3.86	3.86	3.83	3.81	3.85	3.80
Mn2	2.98	2.96	3.00	2.99	2.84	3.01	2.94	2.98
Mn3	3.08	3.02	3.06	3.07	2.84	3.08	2.99	3.05
Mn4	3.74	3.69	3.81	3.76	3.37	3.13	3.22	3.32

Table S8. Calculated QTAIM Mn spin populations for the 0F XFEL models assuming the total charge and spin multiplicity of the S_1 state and for the 1F XFEL models assuming the charge and multiplicity of the S_2 state.

0F	Okayama				Berkeley			
	6JLJ		7COU		6W1O		6DHE	
	A	B	A	B	A	B	A	B
Mn1	3.61	3.62	3.65	3.63	3.73	3.53	3.64	3.59
Mn2	2.74	2.75	2.82	2.78	2.70	2.68	2.74	2.76
Mn3	2.98	2.96	2.96	2.95	2.67	3.04	2.88	2.96
Mn4	3.85	3.85	3.84	3.90	3.59	3.67	3.65	3.68

1F	Okayama				Berkeley			
	6JLK		7CJJ		6W1P		6DHF	
	A	B	A	B	A	B	A	B
Mn1	3.58	3.52	3.59	3.58	3.58	3.56	3.58	3.53
Mn2	2.79	2.78	2.83	2.80	2.66	2.83	2.75	2.79
Mn3	2.91	2.85	2.90	2.91	2.69	2.90	2.81	2.87
Mn4	3.51	3.47	3.58	3.53	3.16	2.94	3.03	3.12

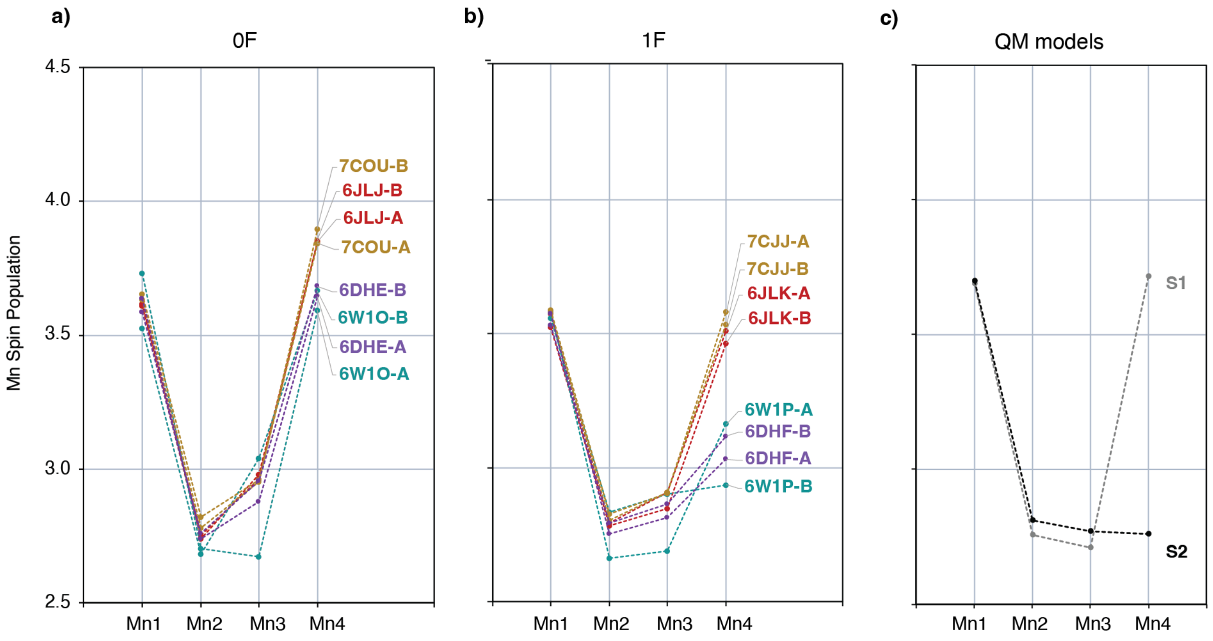

Figure S4. Calculated Mn QTAIM spin populations of: **a)** 0F XFEL models in the S_1 state, **b)** the 1F XFEL models in the S_2 state, and **c)** the QM models in the S_1 and S_2 states.

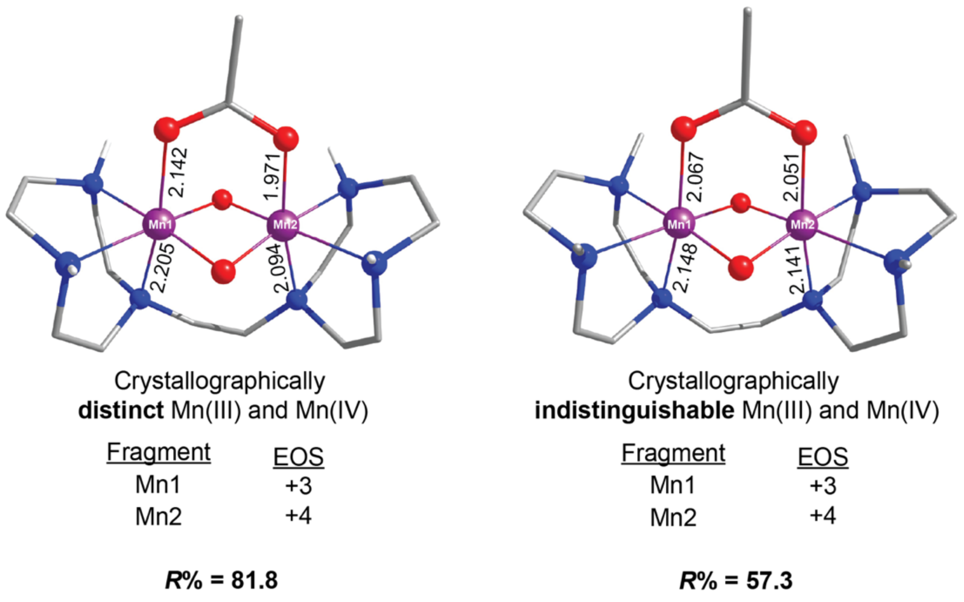

Figure S5. Calculated Mn OSs and the corresponding *R*(%) values derived from EOS analysis of the crystal structures QABHAC (left) and QABGUV (right).[9]

Table S9. *R*(%) values for the EOS calculated using combinations of charge and multiplicity combinations that correspond to different S-states for the 0F and 1F XFEL structures.

0F	Protonation State	6JLJ		7COU		6DHE		6W1O	
	{W1, W2, O4, O5}	A	B	A	B	A	B	A	B
S_0 (III, IV, III, III)	{H_2O, H_2O, OH, O}	**77.6**	**75.4**	67.5	**82.7**	59.7	**81.8**	**76.9**	77.0
	{H_2O, H_2O, O, OH}	73.3	70.6	**72.2**	66.4	**81.0**	80.1	74.1	**81.2**
S_1 (III, IV, IV, III)	{H_2O, H_2O, O, O}	**60.8**	**63.5**	**63.7**	**66.4**	65.9	**59.6**	**77.0**	<50[a]
	{H_2O, OH, O, O}	56.6	59.0	62.9	64.2	65.9	59.4	72.2	<50[a]

1F	Protonation State	6JLK		7CJJ		6DHF		6W1P	
	{W1, W2, O4, O5}	A	B	A	B	A	B	A	B
S_0 (III, IV, III, III)	{H_2O, H_2O, OH, O}	**81.1**	60.5	**79.9**	**77.7**	58.7	60.6	68.4	78.6
	{H_2O, H_2O, O, OH}	77.6	**70.8**	71.3	72.3	**84.5**	**83.5**	**72.7**	**79.0**
S_1 (III, IV, IV, III)	{H_2O, H_2O, O, O}	61.0	75.0	64.7	71.2	69.7	64.4	80.5	66.9
S_2 (III, IV, IV, IV)	{H_2O, H_2O, O, O}	<50[b]	<50[b]	<50[c]	<50[c]	57.4	54.8	55.4	67.6
	{H_2O, OH, O, O}	**51.7**	**58.0**	**56.5**	**58.4**	**66.3**	**64.6**	**68.4**	**71.5**

[a] EOS assignment leads to Mn3(III) and oxyl O5(-1)

[b] EOS assignment leads to Mn4(III) and oxyl O5(-1)

[c] EOS assignment leads to Mn4(III) and oxyl O4(-1)

Table S10. Occupation numbers of frontier EFOs, the last occupied (LO) and first unoccupied (FU) EFOs for the EOS calculations, whose *R*(%) values are given in Table 1 and discussed in the main text.

0F	EFO	6JLJ		7COU		6DHE		6W1O	
		A	B	A	B	A	B	A	B
S_0 (III, IV, III, III)	LO	O5 (0.677)	O5 (0.678)	O4 (0.653)	O2 (0.707)	O2 (0.696)	O2 (0.705)	O5 (0.679)	O4 (0.699)
	FU	Mn4 (0.401)	Mn4 (0.424)	Mn4 (0.431)	Mn4 (0.380)	Mn2 (0.386)	Mn2 (0.387)	Mn3 (0.410)	Mn2 (0.387)
S_1 (III, IV, IV, III)	LO	O5 (0.606)	O5 (0.622)	O5 (0.623)	O5 (0.643)	O5 (0.626)	O5 (0.593)	O5 (0.677)	Mn3 (0.550)
	FU	Mn3 (0.498)	Mn3 (0.487)	Mn3 (0.486)	Mn3 (0.479)	Mn3 (0.467)	Mn3 (0.497)	Mn3 (0.407)	O5 (0.530)

1F	EFO	6JLK		7CJJ		6DHF		6W1P	
		A	B	A	B	A	B	A	B
S_0 (III, IV, III, III)	LO	O5 (0.707)	O4 (0.642)	O1 (0.697)	O2 (0.691)	O4 (0.707)	O2 (0.705)	O2 (0.692)	O2 (0.722)
	FU	Mn4 (0.396)	Mn4 (0.434)	Mn2 (0.398)	Mn2 (0.414)	Mn2 (0.362)	Mn3 (0.370)	Mn3 (0.465)	Mn1 (0.432)
S_1 (III, IV, IV, III)	LO	O5 (0.606)	O5 (0.693)	O5 (0.632)	O5 (0.677)	O5 (0.647)	O5 (0.618)	O2 (0.691)	O1 (0.693)
	FU	Mn3 (0.496)	Mn3 (0.443)	Mn3 (0.485)	Mn3 (0.465)	Mn3 (0.450)	Mn3 (0.474)	Mn2 (0.386)	Mn2 (0.524)
S_2 (III, IV, IV, IV)	LO	W2 (0.617)	O5 (0.656)	O5 (0.656)	W2 (0.657)	O5 (0.652)	O5 (0.667)	O5 (0.674)	O2 (0.679)
	FU	Mn4 (0.600)	Mn4 (0.576)	Mn4 (0.591)	Mn4 (0.573)	Mn4 (0.489)	Mn4 (0.521)	Mn4 (0.490)	Mn3 (0.464)

Table S11. R(%) values for the EOS calculated using combinations of charge and multiplicity that correspond to different S-states for reported QM models of the S_2 state. When EOS assignment does not correspond to the nominal values for the Mn center, the R value is below 50%.

	**Ref. 5**	**Ref. 6**	**Ref. 7**	**Ref. 8**
S_0 (III, IV, III, III)	<50[a]	55.0	<50[b]	<50[b]
S_1 (III, IV, IV, III)	50.2	64.1	63.1	<50[a]
S_2 (III, IV, IV, IV)	**78.4**	**79.4**	**81.0**	**75.8**

[a] EOS assignment leads to Mn2(III) and Mn4(IV)

[b] EOS assignment leads to Mn2(III) and Mn3(IV)

Table S12. Calculated exchange coupling constants J_{ij} (cm^{-1}) and total spins S_{GS} of the ground state (GS) and S_{ES} for the first excited state (ES), and the energy difference ΔE (cm^{-1}) between these spin states.

Model	Mon.	J_{12}	J_{13}	J_{14}	J_{23}	J_{24}	J_{34}	S_{GS}	S_{ES}	ΔE
6W1P	A	-13.54	5.89	23.44	23.65	15.18	12.02	11/2	13/2	3.1
	B	-8.68	6.72	12.50	28.36	4.82	20.21	13/2	11/2	6.5
6DHF	A	-20.30	1.75	29.68	14.99	3.78	-45.70	**1/2**	3/2	45.0
	B	-37.11	-3.51	32.83	13.24	4.56	-34.29	**1/2**	3/2	59.2

Table S13. Spin projection factors, ρ_i, and calculated projected ^{55}Mn hyperfine coupling constants in MHz, for the 6DHF 1F monomers A and B models, and for the QM model of the S_2 state.

Mn ion	Model	ρ_i	DFT			$a_{i,\mathrm{iso}}$	$a_{i,\mathrm{aniso}}$
Mn1	6DHF-A	1.79	-245	-559	-592	-207	84
	6DHF-B	1.67	-242	-528	-583	-201	88
	QM, S_2	1.81	-67	-370	-408	-125	84
Mn2	6DHF-A	–0.99	389	405	410	238	-7
	6DHF-B	-0.97	399	410	431	245	-16
	QM, S_2	-1.00	340	357	385	214	-21
Mn3	6DHF-A	–0.93	351	370	433	228	-43
	6DHF-B	-0.98	318	352	420	216	-50
	QM, S_2	-0.93	301	317	358	193	-29
Mn4	6DHF-A	1.13	-404	-467	-480	-267	26
	6DHF-B	1.28	-390	-440	-482	-259	40
	QM, S_2	1.11	-384	-411	-465	-249	40

Table S14. Experimental and calculated (TPSSh) projected ^{14}N isotropic hyperfine coupling constants (MHz) for the N_{His332} ligating to Mn1.

	$\lvert A_{iso}\rvert$ (MHz)
6DHF-A	2.82
6DHF-B	1.65
QM, S_2	5.98
Exp.[10]	7.1

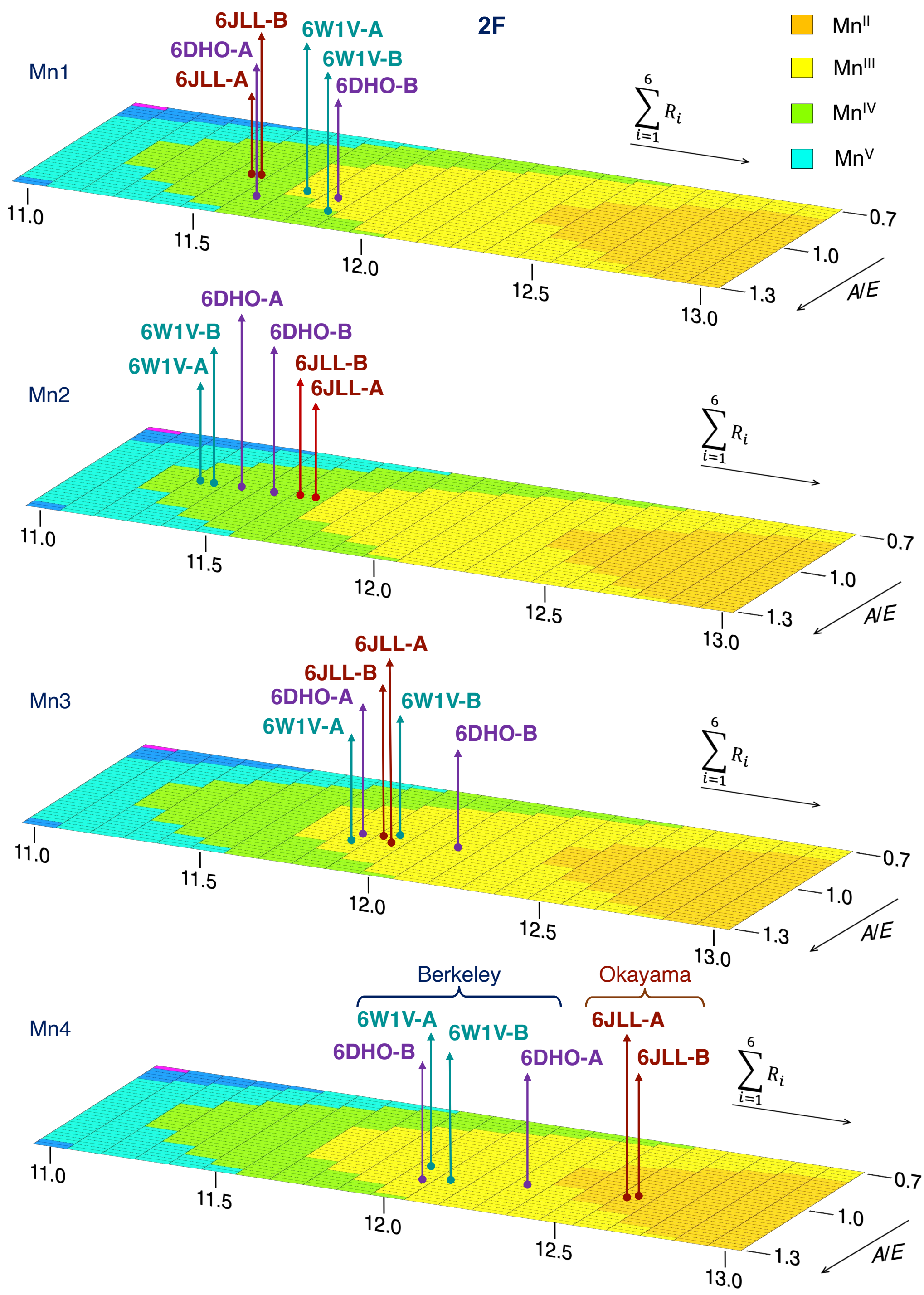

Figure S6. Mn OSs for the 2F XFEL structures derived from bond valence sum analysis using the parameters optimized for Mn(IV).

Table S15. R(%) values and OS assignments by the EOS method, using combinations of charge and multiplicity that correspond to state S_3 for the 2F XFEL structures.

	**O6 protonation**	**Mn1**	**Mn2**	**Mn3**	**Mn4**	**O5**	**O6**	***R*(%)**
**6JLL-A**	**O6H**	IV	IV	IV	**III**	**-1**	-1	69.9
**6JLL-B**		IV	IV	IV	**III**	**-1**	-1	70.6
**6DHO-A**		IV	IV	IV	IV	-2	-1	60.0
**6DHO-B**		IV	IV	IV	IV	-2	-1	63.6
**6W1V-A**		IV	IV	IV	IV	-2	-1	68.7
**6W1V-B**		IV	IV	IV	IV	-2	-1	64.6
**6JLL-A**	**O6**	IV	IV	IV	**III**	-2	**-1**	65.9
**6JLL-B**		IV	IV	IV	**III**	-2	**-1**	64.7
**6DHO-A**		IV	IV	IV	**III**	-2	**-1**	52.2
**6DHO-B**		IV	IV	**III**	**III**	**-1**	**-1**	58.5
**6W1V-A**		IV	IV	IV	**III**	-2	**-1**	56.7
**6W1V-B**		IV	IV	IV	**III**	-2	**-1**	62.0

Table S16. R(%) values for the EOS calculated using combinations of charge and multiplicity that correspond to states S_0, S_1 and S_2 for the 2F XFEL structures.

	**6JLL-A**	**6JLL-B**	**6DHO-A**	**6DHO-B**	**6W1V-A**	**6W1V-B**
S_0[a] (III, IV, III, III)	76.2	78.9	81.7	78.6	79.2	76.6
S_1[a] (III, IV, IV, III)	66.3	70.8	58.8	<50	65.4	66.9
S_2[a] (III, IV, IV, IV)	54.1	<50	60.0	61.6	70.6	62.6

[a]The O6 atom, coordinated to Mn1, was removed from the structures.

References

(1) Suga, M.; Akita, F.; Yamashita, K.; Nakajima, Y.; Ueno, G.; Li, H.; Yamane, T.; Hirata, K.; Umena, Y.; Yonekura, S.; Yu, L.-J.; Murakami, H.; Nomura, T.; Kimura, T.; Kubo, M.; Baba, S.; Kumasaka, T.; Tono, K.; Yabashi, M.; Isobe, H.; Yamaguchi, K.; Yamamoto, M.; Ago, H.; Shen, J.-R. An oxyl/oxo mechanism for oxygen-oxygen coupling in PSII revealed by an x-ray free-electron laser. *Science* **2019**, *366*, 334-338.

(2) Li, H.; Nakajima, Y.; Nomura, T.; Sugahara, M.; Yonekura, S.; Chan, S. K.; Nakane, T.; Yamane, T.; Umena, Y.; Suzuki, M.; Masuda, T.; Motomura, T.; Naitow, H.; Matsuura, Y.; Kimura, T.; Tono, K.; Owada, S.; Joti, Y.; Tanaka, R.; Nango, E.; Akita, F.; Kubo, M.; Iwata, S.; Shen, J.-R.; Suga, M. Capturing structural changes of the S_1 to S_2 transition of photosystem II using time-resolved serial femtosecond crystallography. *IUCrJ* **2021**, *8*, 431-443.

(3) Kern, J.; Chatterjee, R.; Young, I. D.; Fuller, F. D.; Lassalle, L.; Ibrahim, M.; Gul, S.; Fransson, T.; Brewster, A. S.; Alonso-Mori, R.; Hussein, R.; Zhang, M.; Douthit, L.; de Lichtenberg, C.; Cheah, M. H.; Shevela, D.; Wersig, J.; Seuffert, I.; Sokaras, D.; Pastor, E.; Weninger, C.; Kroll, T.; Sierra, R. G.; Aller, P.; Butryn, A.; Orville, A. M.; Liang, M.; Batyuk, A.; Koglin J. E.; Carbajo, S.; Boutet, S.; Moriarty, N. W.; Holton, J. M.; Dobbek, H.; Adams, P. D.; Bergmann, U.; Sauter, N. K.; Zouni, A.; Messinger, J.; Yano, J.; Yachandra, V. K. Structures of the Intermediates of Kok's Photosynthetic Water Oxidation Clock. *Nature* **2018**, *563*, 421-425.

(4) Ibrahim, M.; Fransson, T.; Chatterjee, R.; Cheah, M. H.; Hussein, R.; Lassalle, L.; Sutherlin, K. D.; Young, I. D.; Fuller, F. D.; Gul, S.; Kim, I.-S.; Simon, P. S.; de Lichtenberg, C.; Chernev, P.; Bogacz, I.; Pham, C. C.; Orville, A. M.; Saichek, N.; Northen, T.; Batyuk, A.; Carbajo, S.; Alonso-Mori, R.; Tono, K.; Owada, S.; Bhowmick, A.; Bolotovsky, R.; Mendez, D.; Moriarty, N. W.; Holton, J. M.; Dobbek, H.; Brewster, A. S.; Adams, P. D.; Sauter, N. K.; Bergmann, U.; Zouni, A.; Messinger, J.; Kern, J.; Yachandra, V. K.; Yano, J. Untangling the sequence of events during the $S_2 \rightarrow S_3$ transition in photosystem II and implications for the water oxidation mechanism. *Proc. Natl. Acad. Sci. U. S. A.* **2020**, *117*, 12624-12635.

(5) Krewald, V.; Retegan, M.; Cox, N.; Messinger, J.; Lubitz, W.; DeBeer, S.; Neese, F.; Pantazis, D. A. Metal oxidation states in biological water splitting. *Chem. Sci.* **2015**, *6*, 1676-1695.

(6) Yang, K. R.; Lakshmi, K. V.; Brudvig, G. W.; Batista, V. S. Is Deprotonation of the Oxygen-Evolving Complex of Photosystem II during the $S_1 \rightarrow S_2$ Transition Suppressed by Proton Quantum Delocalization? *J. Am. Chem. Soc.* **2021**, *143*, 8324-8332.

(7) Kawashima, K.; Saito, K.; Ishikita, H. Mechanism of Radical Formation in the H-Bond Network of D1-Asn298 in Photosystem II. *Biochemistry* **2018**, *57*, 4997-5004.

(8) Corry, T. A.; O'Malley, P. J. Proton Isomers Rationalize the High- and Low-Spin Forms of the S_2 State Intermediate in the Water-Oxidizing Reaction of Photosystem II. *J. Phys. Chem. Lett.* **2019**, *10*, 5226-5230.

(9) Schäfer, K.-O.; Bittl, R.; Zweygart, W.; Lendzian, F.; Haselhorst, G.; Weyhermüller, T.; Wieghardt, K.; Lubitz, W. Electronic Structure of Antiferromagnetically Coupled Dinuclear Manganese ($Mn^{III}Mn^{IV}$) Complexes Studied by Magnetic Resonance Techniques. *J. Am. Chem. Soc.* **1998**, *120*, 13104-13120.

(10) Lohmiller, T.; Krewald, V.; Pérez Navarro, M.; Retegan, M.; Rapatskiy, L.; Nowaczyk, M. M.; Boussac, A.; Neese, F.; Lubitz, W.; Pantazis, D. A.; Cox, N. Structure, ligands and substrate coordination of the oxygen-evolving complex of photosystem II in the S_2 state: a combined EPR and DFT study. *Phys. Chem. Chem. Phys.* **2014**, *16*, 11877-11892.
